# The origins and developments of sulfation-prone tyrosine-rich and acidic N- and C-terminal extensions of class ll and lll small leucine-rich repeat proteins shed light on connective tissue evolution in vertebrates

**DOI:** 10.1186/s12862-020-01634-3

**Published:** 2020-06-23

**Authors:** Morten M. Jensen, Henrik Karring

**Affiliations:** Department of Chemical Engineering, Biotechnology and Environmental Technology, University of Southern Denmark, Campusvej 55, 5230 Odense, Denmark

**Keywords:** Extracellular matrix, Collagen, Small leucine-rich repeat protein (SLRP), Tyrosine sulfation, Multiple sequence alignment, Tetrapods

## Abstract

**Background:**

Small leucine-rich repeat protein (SLRP) family members contain conserved leucine-rich repeat motifs flanked by highly variable N- and C-terminal regions. Most class II and III SLRPs have tyrosine-rich N-terminal regions and some of these are sulfated. However, the evolutionary origin and conservation of the tyrosine-rich and acidic terminal regions remain undetermined. In this study, we present the most comprehensive multiple sequence alignment (MSA) analyses of all eight class II and III SLRPs to date. Based on the level of conservation of tyrosine residues and adjacent sequences, we predict which tyrosine residues are most likely to be sulfated in the terminal regions of human class II and III SLRPs.

**Results:**

Using this novel approach, we predict a total of 22 tyrosine sulfation sites in human SLRPs, of which only 8 sites had been experimentally identified in mammals. Our analyses suggest that sulfation-prone, tyrosine-rich and acidic terminal regions of the class II and III SLRPs emerged via convergent evolution at different stages of vertebrate evolution, coinciding with significant evolutionary events including the development of endochondral bones and articular cartilage, the aquatic to terrestrial transition, and the formation of an amnion.

**Conclusions:**

Our study suggests that selective pressures due to changes in life conditions led to the formation of sulfotyrosine-rich and acidic terminal regions. We believe the independent emergence and evolution of sulfotyrosine-rich and acidic N- and C-terminal regions have provided each class II and III SLRP member with novel vital functions required to develop new specialized extracellular matrices and tissues in vertebrate species.

## Background

**Fig. 1:**
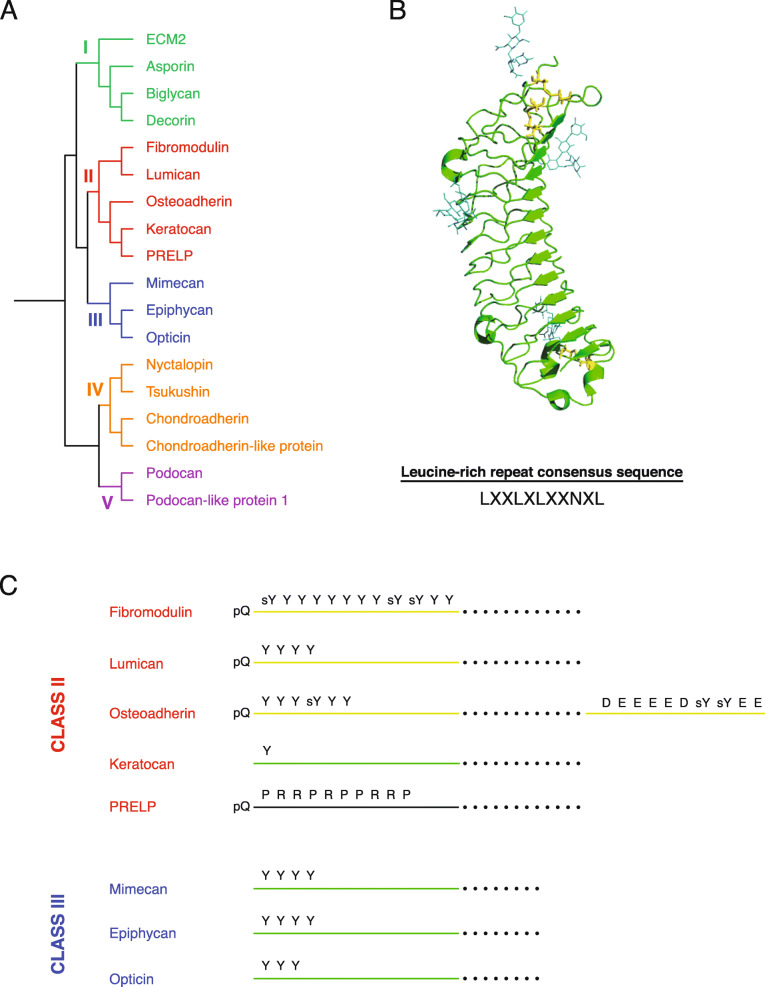
Phylogenetic analysis of human SLRPs and schematic representation of class II and III SLRPs. **a** The rooted dendrogram shows the phylogenetic relationship between the 18 known human SLRPs with colours displaying the five classes of SLRPs. **b** Crystal structure of fibromodulin (PDB: 5MX0). Open-Source PyMOL was used for presenting the crystal structure. The fibromodulin structure contains several sugar moieties (dark green) and three disulfide-bridges formed between cysteine residues (yellow) in the N- and C-terminal cysteine motifs. The N-terminus is pointing upwards. Note that the variable N-terminal region of fibromodulin is not visible in the structure due to its disordered structure. The leucine-rich repeat consensus sequence (LXXLXLXXNXL, where L is leucine or another hydrophobic residue, N is asparagine and X is any residue) is shown. The repetition of this motif gives the SLRPs their topology with parallel β-strands on the inner concave face and a variable structure on the outer convex face resulting in an overall curved solenoid structure. **c** Schematic representation of selected elements in the terminal regions of human class II and III SLRPs. The dots in the dashed lines represent leucine-rich repeats, while solid lines represent the N- and C-terminal regions of the SLRPs**.** Yellow lines represent termini experimentally known to be sulfated, while the green lines represent termini predicted to be sulfated according to current literature. The letter abbreviations denote the following features: “**pQ**” indicates an N-terminal glutamine which in vivo will cyclize into pyroglutamate (pQ); “**Y**” represent tyrosines; “**sY**” are tyrosine sites for which sulfation has been identified experimentally in humans; “**D**” and “**E**” represent the aspartic and glutamic acid residues clustered in the C-terminal region of osteoadherin; “**P”** and “**R**” represent the prolines and arginines in the N-terminal region of PRELP

During the evolution of vertebrate species, several novel connective tissues and organs have evolved with important functions for the organisms. Some of these specialized tissues are endochondral bones, the lung (or swim bladder), the amnion and the placenta. Genes encoding extracellular matrix (ECM) proteins have been modified, duplicated or developed de novo to create these tissues [1]. One important family of ECM proteins is the small leucine-rich repeat protein (SLRP) family. The SLRP family consists of 18 known extracellular proteins in humans which are found ubiquitously in the body, but different SLRPs are expressed in different specialized extracellular matrices [2]. SLRPs generally have roles in collagen binding, modifying collagen fibrillogenesis and growth factor and cytokine signalling regulation, but each SLRP also has unique functions [2–5]. Most members of the SLRP family can carry one or more glycosaminoglycan (GAG) chains, but the pattern of GAGs varies between tissues or stages of life for some SLRPs [3, 5]. SLRPs contain several leucine-rich repeat motifs and conserved N- and C-terminal cysteines [6, 7]. The cysteine residues in the N-terminus are found in different cluster motifs; the cluster motifs together with the number of leucine-rich repeats, evolutionary conservation, and genomic organization have been used to divide the SLRPs into five classes (Fig. 1a) [3, 4, 6, 7]. The class I SLRPs decorin and biglycan are characterised by containing a pro-peptide functioning as a recognition signal for modifying the proteins by addition of GAG chains. Members of class II SLRPs are characterised by containing clusters of sulfotyrosine residues in their N-terminal region and can carry keratan sulfate and polylactosamine chains. Class III SLRP members have the lowest number of leucine-rich repeats and they are often found as proteolytically processed variants [3, 7–10]. Class IV contains the non-canonical SLRPs, which are more structurally diverse from each other than those in the other classes. Yet, the class IV SLRPs appear to share an evolutionary origin [1]. The two class V SLRPs have not been thoroughly characterised but podocan share properties such as collagen binding with the other SLRPs [11]. Crystal structures of SLRPs, e.g. fibromodulin, reveal a common structural fold that resembles other proteins with leucine-rich repeats. Thus, the SLRP structure has the form of a curved solenoid with a long β-beta sheet on the concave side with each of the leucine-rich repeats contributing to one β-strand, while the convex side has a variety of secondary structure elements (Fig. 1b) [12]. According to previous studies, SLRPs appear to have emerged in early chordates. For example, the sea squirt (*Ciona intestinalis*) has three SLRP-encoding genes, one of these being the orthologue of the ancestral gene to all extant class I, II, and III SLRP genes [1, 6]. The N- and C-terminal regions (terminal sequences beyond the conserved cysteine clusters) of the SLRPs are highly variable between the SLRPs. The variable terminal regions provide specific functions to each SLRP through different structural elements such as clusters of tyrosines or acidic or basic amino acid residues and attached GAG chains or other polysaccharides. Specifically, tyrosine clusters are present exclusively in class II and III SLRPs (Fig. 1c) [2, 5].

Tyrosine sulfation is a post-translational modification (PTM) in which a sulfonate group from the donor 3′-phosphoadenosine 5′-phosphosulfate (PAPS) is transferred to the hydroxyl group of a peptidyltyrosine residue. The sulfation reaction is catalysed by tyrosylprotein sulfotransferase 1 or 2 [13]. Sulfotyrosine (sTyr, sY) residues are generally known to mediate protein-protein interactions and are critical in a diverse range of physiological functions [13–15]. Up to 1% of the total number of tyrosine residues in metazoan proteins is estimated to be sulfated [16], and on average, one out of three proteins secreted by fibroblasts contains a sulfotyrosine residue [17]. Despite this relatively high prevalence of tyrosine sulfation, both the precise position and biological function of sulfotyrosines in many extracellular proteins remain unknown. This is mainly due to lability of tyrosine sulfation under standard analysis methods [13] and because there is no universal consensus sequence requirement for sulfation of tyrosine in proteins. However, several common features of residues neighbouring tyrosines have been determined experimentally and in silico as important for tyrosine sulfation (see Table 2 in the Methods section) [13–15, 18–20]. Secondary structure also plays a major role in the sulfation of tyrosines as an intrinsically unfolded conformation around the acceptor tyrosine stimulates sulfation. However, there are examples of tyrosine-sulfated proteins that deviate from some of these sulfation determinants [14, 15, 19], which may reflect that unfavourable features can be compensated for by other elements stimulating tyrosine sulfation. Software tools for prediction of tyrosine sulfation have been developed, but their success rates remain very moderate [13, 21]. Thus, in a previous study, software-based prediction of tyrosine sulfation in SLRPs was not accurate [22].

Except for proline-arginine-rich end leucine-rich repeat protein (PRELP), also known as prolargin, all class II and III SLRPs have been predicted or shown to contain tyrosine sulfations (Fig. 1c) [2, 22–24]. For fibromodulin and lumican, it has been experimentally determined that sulfations are found in tyrosine clusters in the N-terminal region of the proteins. For osteoadherin (also called osteomodulin), a cluster of sulfotyrosines is found in the N-terminal region, while two adjacent sulfotyrosine residues are present in the C-terminal region. Likewise, for the remaining class II and III SLRPs, sulfotyrosines have been predicted in the N-terminal regions. However, only the functions of the sulfotyrosine-rich N-terminal region of fibromodulin has been unravelled. The sulfotyrosine-rich and acidic N-terminal region of mammalian fibromodulin acts as a GAG-mimic and consequently has the ability to interact with several heparin-binding proteins and bioactive factors in vitro [25]. In addition, a peptide consisting of the sulfotyrosine-rich N-terminal region of fibromodulin binds collagen and enhances fibrillogenesis in vitro, while full-length fibromodulin inhibits fibrillogenesis [26]. Furthermore, this sulfated peptide directs the formation of highly organized collagen fibrils. Thus, the sulfotyrosine cluster gives fibromodulin the potential to bind two different collagen molecules simultaneously, which may be relevant for fine-tuning collagen fibrillogenesis. Knowledge about the molecular evolution and development of the highly variable N- and C-terminal regions of SLRPs is limited; therefore, comparison of SLRP sequences from species of different animal classes will shed light on the evolution of this important family of ECM proteins in relation to their functional roles in different types of connective tissues. In this study, we retrieved and rigorously assessed amino acid sequences for each of the eight class II and III SLRP family members. After the assessment, 527 sequences from different jawed vertebrate species remained, and these sequences were aligned and analysed with special focus on the N- and C-terminal regions. The N-terminal regions, comprised of the sequence preceding the first conserved cysteine residue, revealed different levels of variation through the evolution of each SLRP. The largest and most significant differences were found in fibromodulin, osteoadherin and mimecan, in which acidic tyrosine clusters appear to have emerged independently in the N-terminal regions at different points in vertebrate and tetrapod (*Tetrapoda*) evolution; this suggests convergent evolution of (sulfo)tyrosine-rich terminal sequences. The results indicate that the development of acidic and sulfation-prone tyrosine-rich terminal regions of the SLRPs coincided with significant evolutionary events and changes in connective tissues including the development and modification of bone tissue, articular cartilage and the amnion. Therefore, this study sheds light on the evolution of vertebrates and tetrapods as well as the functional roles of the N- and C-terminal regions of class II and III SLRPs in connective tissues. In addition, based on the degree on conservation of tyrosine residues and the adjacent features facilitating tyrosine sulfation, we predict specific tyrosine residues in the terminal regions of SLRPs which are likely to be sulfated.

## Results

### Phylogenetic analysis of class II and III SLRP members

The phylogenetic analyses show that the sequences of each member of class II and III SLRPs between species are grouped together but clearly separated from the other SLRP class members (Additional file 1**Fig. S1**). This confirms that the sequences were correctly annotated and categorized in the SLRP datasets used in the study. However, four sequences annotated as lumicans from different orders of ray-finned fish (*Actinopterygii*) are rooted before the split between lumican and fibromodulin (Additional file 1**Fig. S1A**). These lumican/fibromodulin-like sequences cannot be grouped with either lumican or fibromodulin and were therefore not included in any of the datasets. The four sequences are denoted as “unknown class II SLRP” in Fig. S1A. In addition, one sequence (>tr|A0A0P7UKK4|A0A0P7UKK4_9TELE) annotated as an epiphycan is rooted together with opticin sequences and was therefore transferred to the opticin dataset (Additional file 1**Fig. S1B**).

### Class II SLRPs

#### An internal tyrosine-rich sequence in the N-terminal region of fibromodulin developed after the amphibian-amniote split

**Fig. 2:**
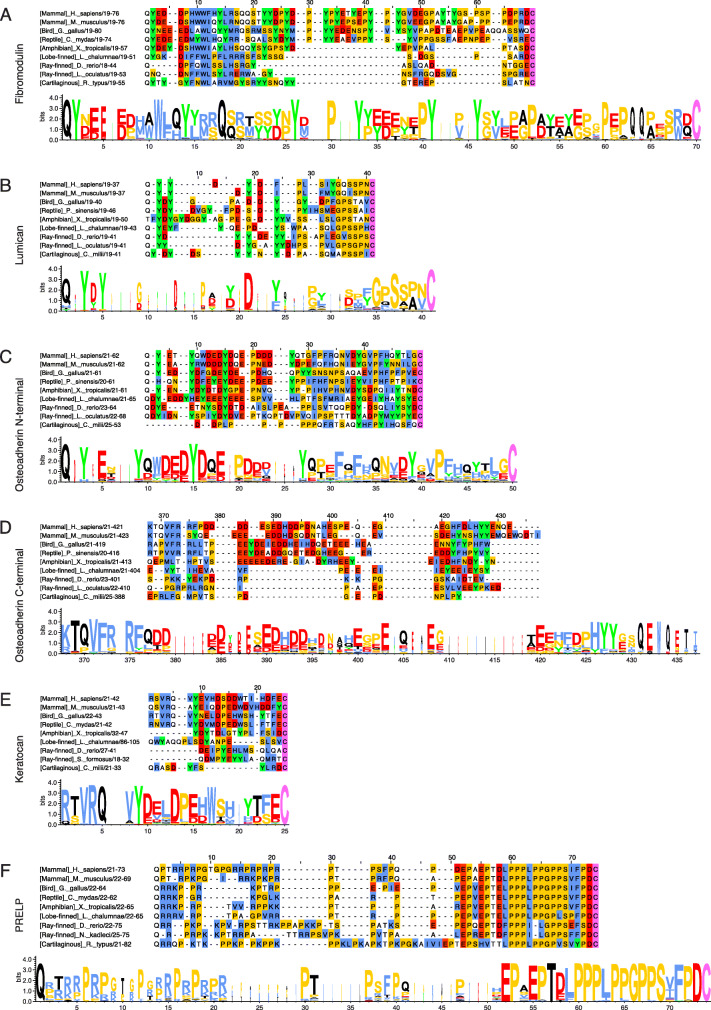
Alignments of class II SLRPs reveal the molecular evolution of their terminal regions. The upper panel in each subfigure contains representative sequences of the N-terminal regions of class II SLRPs from the class II SLRP MSAs (Additional file 1) starting from the first amino acid (position 1) after the signal peptide and ending at the first conserved cysteine. For the osteoadherin C-terminal region, the area starting from position 368 to the end of the MSA at position 437 is represented. Lower panels contain sequence logo representations of the N- or C-terminal regions of each class II SLRP. Description of the residue colour coding can be found in the methods section. **a:** Fibromodulin representative sequences and sequence logo of amniote sequences (63 sequences). **b:** Lumican representative sequences and sequence logo of jawed vertebrate sequences (95 sequences). A degree of variation in the amino acid composition and length of the N-terminal region of lumican in different taxonomic genera is evident from the gaps introduced in the MSA. **c:** Representative sequences of the N-terminal region of osteoadherin and sequence logo of bony vertebrate sequences (38 sequences). **d:** Representative sequences of the C-terminal region of osteoadherin and sequence logo of tetrapod sequences (34 sequences). **e:** Keratocan representative sequences and sequence logo of tetrapod sequences (55 sequences)**. f:** PRELP representative sequences and sequence logo of jawed vertebrates sequences (39 sequences). The representative sequences are from *Homo sapiens* (Q06828; P51884; Q99983; O60938; P51888), *Mus musculus* (P50608; P51885; O35103; O35367; Q9JK53), *Gallus gallus* (P51887; P51890; R4GF52; O42235; A0A1D5PAN0), *Chelonia mydas* (M7AZ87; M7BEH4; XP_007065190.1), *Pelodiscus sinensis* (K7F6Y3; K7G746), *Xenopus tropicalis* (F6RIJ3; Q640B1; XP_012817254.1; XP_002937114.2; A4IIL0), *Latimeria chalumnae* (XP_006002318.1; H2ZW54; XP_005987119.1; XP_014352190.1; H3ADS3), *Danio rerio* (F1QG51; Q6IQQ7; F6NL91; Q5RI43; F1QY29), *Lepisosteus oculatus* (W5N2Q9; W5NHY1; W5N8Y0), *Scleropages formosus* (A0A0P7VL56), *Nothobranchius kadleci* (A0A1A8BTR3), *Rhincodon typus* (XP_020392147.1; XP_020368858.1), and *Callorhinchus milii* (XP_007893500.1; V9NEN7; XP_007893501.1)

By observing the MSA of fibromodulin, it is evident that the N-terminal region is extended with an internal tyrosine-rich sequence (positions 30–64) in amniotes (reptiles, birds, and mammals) (Fig. 2a, Additional file 1 Fig. S2 and Table S1). However, while it does not have an extended N-terminal region such as amniotes, the number of tyrosines in the N-terminal region of fibromodulin from whale shark, a cartilaginous fish (*Chondrichthyes*), is relatively high (Additional file 1**Table S1**). Up to six and nine tyrosine sulfations are known in the N-terminal region of human and bovine fibromodulin, respectively [22]. It is noteworthy that 9–12 tyrosines are found in the N-terminal region of all amniote fibromodulins, indicating that species of this clade potentially have the same number of tyrosine sulfation sites as human and bovine fibromodulin. Analysis of the sequence logo of the N-terminal region of amniote fibromodulin sequences reveals that tyrosines at positions 2, 26, 33, 41, and 46 are highly conserved in amniotes (100, 100, 98, 100 and 97%, respectively) (Fig. 2a). It also shows a large degree of conservation of amino acids favouring tyrosine sulfation (read the Introduction and Table 2 in the Methods section for information about features favouring tyrosine sulfation). Thus, with the exception of the tyrosines at positions 14–15, all the tyrosines in the N-terminal region (positions 2, 22–24, 26, 33–35, 38, 41, 46, 48, 54, and 56) have proximal sequences favouring sulfation which explains their conservation in amniote evolution. Examining the primary structural features in the shorter N-terminal region of fibromodulin from *Xenopus tropicalis* also reveals sulfation-favourable characteristics for some of its tyrosine residues. An N-terminal glutamine (Q) is highly conserved (90%) in all jawed vertebrates (*Gnathostomata*), suggesting that a pyroglutamate (pQ) at position 1 in fibromodulin is crucial in all animal classes studied with the exception of some ray-finned fish (Additional file 1**Fig. S2**). Hence, a tyrosine-rich N-terminal extension with structural features promoting tyrosine sulfation is clearly present in all amniote fibromodulins, while the tyrosine-rich N-terminal region of fibromodulin is shorter and less profound or absent in anamniotes (amphibians, lobe-finned fish (*Actinistia*), ray-finned fish, and cartilaginous fish) (Fig. 2a **and** Additional file 1**Fig. S2**).

#### A tyrosine-rich N-terminal region with features promoting tyrosine sulfation is present in lumican of jawed vertebrates

It is known that lumican from human and cow has two tyrosine sulfations in the N-terminal region; however, the precise tyrosines carrying these modifications are unknown [22], while all four tyrosines in the N-terminal region of lumican from mouse have been identified as sulfated [23]. According to the MSA of lumican, all jawed vertebrates have lumican with a tyrosine-rich N-terminal region and some variations in length and amino acid composition (Fig. 2b**,** Additional file 1**Fig. S3 and Table S1**). All jawed vertebrate lumicans have an N-terminal region of 18–31 residues containing 2–6 tyrosines (Additional file 1**Table S1**), which suggests that the N-terminal region of lumican has not adapted novel functions during the evolution of the jawed vertebrate species. Conservation of tyrosines (e.g., positions 3 (95%) and 5 (78%)) and concomitant features facilitating tyrosine sulfation in the N-terminal region of jawed vertebrate lumicans are evident (Fig. 2b). Thus, in addition to tyrosines and the N-terminal glutamine at position 1, several residues including D, P, G and S are rather predominant and conserved (Fig. 2b). Therefore, sulfation of tyrosines in the N-terminal region of lumican appears to be conserved throughout all extant jawed vertebrates.

#### An acidic and tyrosine-rich N-terminal extension is present in osteoadherin from bony vertebrates

Up to eight tyrosine sulfations have been identified in recombinant human osteoadherin containing an extra N-terminal tyrosine. Six of the sulfotyrosines were found in the N-terminal region, and two were present in the C-terminal region of osteoadherin [22]. From the MSA, it is observed that the N-terminal region of osteoadherin from bony vertebrates (*Euteleostomi*) is extended 7–18 residues compared to osteoadherin in cartilaginous fish (*Chondrichthyes*) (Fig. 2c**,** Additional file 1**Fig. S4 and Table S1**). This extension of the N-terminal region is highly acidic (position 1–28) and contains four conserved tyrosines at positions 3, 9, 15, and 27 (92, 89, 79 and 79%, respectively). Beyond the extension (positions 29–49), the features generally appear less favourable for tyrosine sulfation. However, the tyrosine at position 39 is also highly conserved (79%) and flanked by several residues promoting sulfation. Additionally, an N-terminal glutamine, and consequently a pyroglutamate, is conserved in osteoadherin across jawed vertebrates with the exception of the ghost shark (*Callorhinchus milii*). The presence of an acidic and tyrosine-rich N-terminal extension of osteoadherin in extant bony vertebrates indicates that an N-terminal sulfotyrosine cluster is conserved throughout this clade but is absent in cartilaginous fish.

#### The acidic C-terminal extension is only present in osteoadherin from tetrapods

Osteoadherin’s large acidic C-terminal region is a unique feature among the SLRPs. However, the MSA of osteoadherin reveals that this feature is only present in tetrapods (Fig. 2d**,** Additional file 1**Fig. S5 and Table S1**). Thus, the C-terminal region of osteoadherin from tetrapods is extended 12–51 residues and contains many acidic residues (position 378–421) compared to osteoadherin from lobe-finned (i.e., *Latimeria chalumnae*), ray-finned and cartilaginous fish (Fig. 2d**,** Additional file 1**Fig. S5 and Table S1**). The number of tyrosines in the C-terminal region of osteoadherin is also generally higher in tetrapods than in ray-finned and cartilaginous fish (Additional file 1**Table S1**). One or more tyrosines in the C-terminal region of all jawed vertebrate osteoadherins is located in sequences favouring sulfation. Tyrosine sulfation of the C-terminal region of osteoadherin could therefore be conserved throughout the evolution of jawed vertebrate species. Specifically, the C-terminal region of osteoadherin from tetrapods contains two highly conserved tyrosines at positions 427 and 428 (79 and 82%, respectively) (Fig. 2d), which are sulfated in recombinant human osteoadherin [22].

#### A single, highly conserved tyrosine and several features promoting tyrosine sulfation are present in the N-terminal region of keratocan of tetrapods

The MSA of keratocan shows that the N-terminal region of keratocan from amniotes extends 6–10 residues compared to keratocan in amphibians, and ray-finned and cartilaginous fish. The lobe-finned fish has an N-terminal region close in length to amniote keratocans (Fig. 2e**,** Additional file 1**Fig. S6 and Table S1**). However, in all jawed vertebrates, the N-terminal region of keratocan contains 1–3 tyrosines (Additional file 1**Table S1**). Sequence logo analysis of the N-terminal region of keratocan from tetrapods reveals that only the tyrosine at position 9 is highly conserved (98%), but the tyrosine has proximal amino acid residues both facilitating and suppressing tyrosine sulfation (Fig. 2e). Thus, several features favourable for tyrosine sulfation are found C-terminally, while unfavourable features are located N-terminally to the tyrosine at position 9 in tetrapod keratocans. Hence, based on the conservation of at least one tyrosine and sulfation-favourable features adjacent to the tyrosine, if sulfation is confirmed in one species, it is plausible that the modification is conserved in all tetrapods. Nevertheless, tyrosines and sulfation-favourable features are also found in the other classes; therefore, tyrosine sulfation appears possible for these animals as well.

#### PRELPs from jawed vertebrates all contain a proline/basic residue-rich N-terminal region

PRELP does not contain a tyrosine-rich N-terminal region like the other class II SLRPs, but instead it has an N-terminal region rich in prolines and arginines. No major differences in the N-terminal region of PRELP can be observed in jawed vertebrate species (Fig. 2f **and** Additional file 1**Fig. S7**). However, there are variations in the N-terminal length and more lysines than arginines in some animals (Additional file 1**Table S2**). There is a slight tendency for the ratio of arginines to lysines to shift towards more arginines from cartilaginous fish towards mammals. Additionally, there is variation in the number of prolines and arginines/lysines from organism to organism. Notably, the N-terminal residue after the signal peptide is a completely conserved glutamine. Thus, the sequence analysis indicates that no specific N-terminal sequence is required to maintain PRELP’s N-terminal function as long as it contains a certain minimum repetitive sequence of proline and arginine/lysine residues.

### Class III SLRPs

#### A tyrosine-rich N-terminal region of mimecan with features promoting tyrosine sulfation evolved in sarcopterygians

**Fig. 3:**
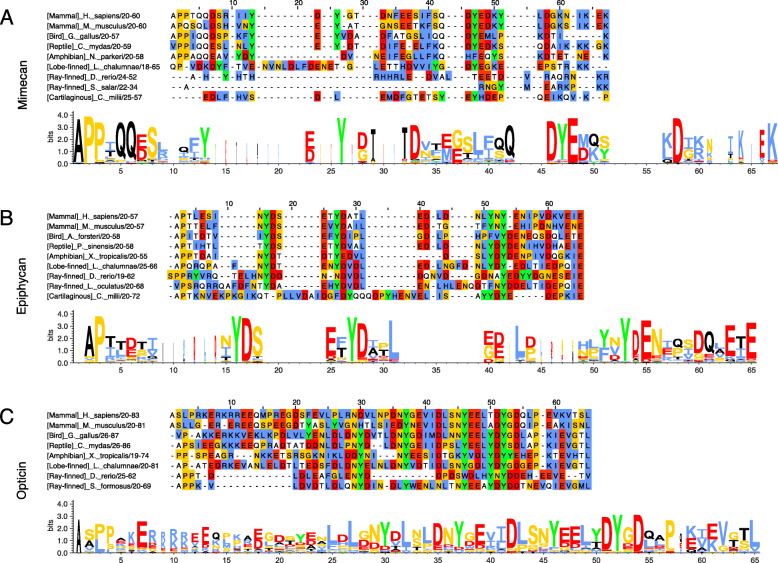
Alignments of class III SLRPs reveal the molecular evolution of their N-terminal regions. The upper panel in each subfigure is representative of sequences of the N-terminal regions of class III SLRPs from the class III SLRP MSAs (Additional file 1) starting from the first amino acid (position 1) after the signal peptide and ending at position 67 for mimecan and 65 for epiphycan and opticin. Lower panels are sequence logo representations of the N-terminal regions of each class III SLRP. Description of the residue colour coding can be found in the methods section. **a:** Mimecan representative sequences and sequence logo of tetrapod sequences (74 sequences). A large degree of variation in the length and amino acid composition of the N-terminal region of mimecan is evident from the number of gaps introduced in the MSA **b:** Epiphycan representative sequences and sequence logo of bony vertebrates sequences (77 sequences). Short extensions of the N-terminal region are found in ray-finned and cartilaginous fish at positions 9–14 and positions 19–24 and 32–39 in ghost shark epiphycan. **c:** Opticin representative sequences and sequence logo of bony vertebrates sequences (22 sequences). In lobe-finned fish and tetrapods the N-terminal region of opticin is extended with a sequence containing both basic and acidic residues (positions 8–19) compared to that in ray-finned fish. The representative sequences are from *Homo sapiens* (P20774; Q99645; Q9UBM4), *Mus musculus* (Q62000; P70186; Q920A0), *Gallus gallus* (Q9W6H0; Q6YEX8), *Aptenodytes forsteri* (A0A087RBX5), *Chelonia mydas* (M7CFR9; XP_007065189.1), *Pelodiscus sinensis* (K7F6Z9), *Nanorana parkeri* (XP_018407801.1), *Xenopus tropicalis* (Q6DK81; Q28HP1), *Latimeria chalumnae* (XP_005987115.1; XP_006009490.1; XP_006002321.1), *Danio rerio* (Q1LV51; Q5RI46; Q15JE7), *Salmo salar* (B5X6F3), *Lepisosteus oculatus* (W5NHX7), *Scleropages formosus* (A0A0P7UKK4), and *Callorhinchus milii* (V9L0I5; XP_007893502.1)

The MSA of mimecan reveals variability in the N-terminal region both within classes and between different classes (Fig. 3a**,** Additional file 1**Fig. S8 and Table S3)**. Additionally, it shows that tetrapod and lobe-finned fish mimecans generally contain 3–4 tyrosines in the N-terminal region, while 1–2 tyrosines are present in the N-terminus of ray-finned and cartilaginous fish. The sequence logo analysis of the N-terminal region of tetrapod mimicans revealed that they have highly conserved tyrosines at positions 26 and 47 (88 and 89%, respectively) with adjacent and concomitant features facilitating tyrosine sulfation including acidic and turn-inducing residues (Fig. 3a). Tyrosine at position 13 is also rather conserved in tetrapods (80%). One or two tyrosines with local sequences favouring sulfation are present in some species of lobe-finned, ray-finned and cartilaginous fish, e.g.*,* positions 42 and 47 in sharks, while they are not present in others such as zebrafish (Fig. 3a **and** Additional file 1**Fig. S8**). Overall, this implies the possibility of a conserved cluster of sulfotyrosines in the N-terminal region of mimecan in tetrapods and lobe-finned fish but does not exclude the presence of few sulfotyrosines in mimecans of some species of fish.

#### Highly conserved tyrosines and features promoting tyrosine sulfation are present in the N-terminal region of epiphycan from bony vertebrates

The lengths of the N-terminal regions of epiphycans are nearly the same for all classes, but some species of ray-finned fish have epiphycans with N-terminal regions close to double the length of the others (Fig. 3b, Additional file 1**Fig. S9 and Table S3**). All jawed vertebrates have epiphycans with an N-terminal region containing tyrosines. The N-terminal region of bony vertebrate epiphycans contains 3–7 tyrosines (Additional file 1**Table S3**), and the tyrosines at positions 16, 27, and 53 are highly conserved (99, 97 and 96%, respectively) (Fig. 3b). All three conserved tyrosines have acidic residues in their + 1 positions and additional acidic- and turn-inducing residues in their surrounding residues which promote tyrosine sulfation (Fig. 3b). Thus, the potential for sulfation of several tyrosines in the N-terminal region of epiphycan appears to be conserved throughout all extant bony vertebrates. However, residues promoting sulfation can also be observed in connection with tyrosines in the N-terminal region of cartilaginous fish epiphycans (Fig. 3b), thus substantiating the possibility of tyrosine sulfation in all jawed vertebrates.

#### Tyrosines with sulfation-promoting amino acids in their proximity are conserved in the N-terminal region of opticin in bony vertebrates

Human opticin is known to be tyrosine-sulfated, but the specific site(s) of sulfation remain unknown [24]. However, it has been suggested that tyrosine sulfations in opticin are located in the N-terminal region as observed for the other class II and III SLRPs. The MSA reveals that the N-terminal regions of all bony vertebrate opticins are approximately the same length and contain 2–6 tyrosines; Chinese tree shrew (*Tupaia chinensis*) is the one exception as it has no tyrosines (Fig. 3c, Additional file 1**Fig. S10 and Table S3**). Sequence logo analysis of the N-terminal region of bony vertebrate opticins shows conserved tyrosines at positions 30, 37, 46 and 52 (77, 77, 82 and 95%, respectively). In addition, these four conserved tyrosines have concomitant adjacent residues promoting tyrosine sulfation (acidic and turn-inducing residues with some aliphatic residues) (Fig. 3c). The conservation of tyrosines and sulfation-favouring features in the N-terminal region of bony vertebrate opticin supports the suggested N-terminal location of tyrosine sulfation(s) in human opticin.

## Discussion

SLRPs play essential roles in the development and homeostasis of extracellular matrices in connective tissues of vertebrates. In the present study, we used multiple sequence analyses to compare the SLRP sequences from different animal species representing different taxonomic classes for each of the eight class II and III SLRPs. With special focus on the highly variable tyrosine-rich and acidic N- and C-terminal regions, we identified specific tyrosine residues and acidic regions in different SLRPs which are highly conserved in different taxonomic classes. Using sequence logo analysis, we show that residues promoting tyrosine sulfation flank most of the conserved tyrosine residues.

### Prediction of tyrosine sulfation sites based on conservation of tyrosines and adjacent residues

Experimental determination of tyrosine sulfation is hindered by the lability of the modification in standard analysis methods. Therefore, few tyrosine sulfation sites have been determined in class II and III SLRPs (Fig. 1**and** Table 1). It is reasonable to believe that sulfated tyrosines, which mediate specific protein-protein interactions, are more conserved than non-sulfated tyrosines. Thus, an in silico analysis of tyrosine-rich sequences and surrounding consensus features influencing tyrosine sulfation could possibly be used to predict the existence of sulfotyrosines. Our MSA and sequence logo analyses revealed that conserved tyrosine residues with consensus features promoting tyrosine sulfation are present in all analysed SLRPs from animal species having a tyrosine-rich N-terminal region. Consequently, this suggests that sulfotyrosines are present in all of these SLRPs. This opens for the possibility of predicting sulfation sites based on the degree of conservation of tyrosines and the adjacent residues promoting or suppressing tyrosine sulfation. Thus, at this point in the study we want to predict which of the tyrosines present in the human sequences are prone to be sulfated based on conservation of tyrosines and surrounding residues known to stimulate sulfation. In accordance with the results from the MSAs we focused on predicting which tyrosine residues in the N- and C-terminal regions of human class II and III SLRPs are most likely to be sulfated. Using this novel approach, we have predicted a total of 22 tyrosine sulfation sites in human SLRPs, of which only 8 sites have been experimentally identified in mammals (Table 1). Notably, the six tyrosine sulfation sites experimentally identified in human class II and III SLRPs in previous studies [22, 23] are all predicted to be sulfated (Table 1). Thus, the predictions are correct for the identified N-terminal sulfotyrosine and for both C-terminal sulfotyrosines in human osteoadherin. Our approach also correctly identified three confirmed sulfotyrosines known in human and bovine fibromodulin and predicted an additional two tyrosine sulfation sites in the middle of the N-terminal tyrosine-rich region in fibromodulin. For lumican, the approach successfully identified two out of four known sulfation sites which have previously been experimentally confirmed in mouse lumican (Table 1). Hence, this approach is useful in predicting tyrosine residues which are likely to be sulfated. However, it may have limitations similar to those of tyrosine sulfation prediction software. According to observations by V. Tillgren et al. [26], the number of sulfotyrosines are more important for the function of a sulfotyrosine cluster than the specific position of the modifications in the cluster. Therefore, some tyrosines in the clusters may move a few positions through evolution without affecting the functionality of the sulfotyrosine cluster. This makes it difficult to successfully predict all specific sulfotyrosine residues. E.g., the novel approach failed to predict the tyrosines in MSA and sequence logo positions 19 and 34 in lumican as sulfated (Fig. 2b) although they have been experimentally identified as sulfotyrosines in mouse (Table 1). Additionally, it is essential to choose only relevant phylogenetic classes for the prediction approach. Thus, only the classes containing the conserved cluster(s) of tyrosines within a lineage according to the MSA are included in the logo analysis. Adding sequences from phylogenetic classes without conserved tyrosines will simply distort the prediction. A strength of the presented prediction approach is that if a sulfation site is known in one organism it is likely to be present in another if the tyrosine residue is conserved. In contrast to tyrosine sulfation prediction software available today, this novel approach includes a conservation assessment of tyrosines and adjacent residues between classes of animals, which reflects the importance of the identity of the residues. Therefore, one can predict tyrosine sulfations in proteins from less-studied species using sequences of the same protein from other related and well-studied organisms. We propose that data on conservation of tyrosines and adjacent residues could be integrated into software for tyrosine sulfation prediction, thereby improving the rate of successful prediction.

**Table 1:** Tyrosine sulfation site prediction

Class	SLRP (animals)	Positions of predicted tyrosine sulfation sites in sequence alignment and logo (Fig. no.)	Corresponding tyrosine residues in precursor human SLRP	Experimentally confirmed tyrosine sulfation sites in precursor SLRP (species)	References
II	Fibromodulin (amniotes)	2 (Fig. 2)	20	20 (human) / 20 (bovine)	[22]
				38 (bovine)	
		26 (Fig. 2)	42		
		33 (Fig. 2)	45		
		41 (Fig. 2)	53	53 (human) / 53 (bovine)	
		46 (Fig. 2)	55	55 (human) / 55 (bovine)	
				63 (bovine)	
				65 (bovine)	
II	Lumican (jawed vertebrates)	3 (Fig. 3)	20	20 (mouse)	[22, 23]
		5 (Fig. 3)	21	21 (mouse)	
				23 (mouse)	
				30 (mouse)	
II	Osteoadherin (bony vertebrates (N-terminal); tetrapods (C-terminal))	3 (Fig. 4)	22		[22]
		9 (Fig. 4)	25		
		15 (Fig. 4)	31		
		27 (Fig. 4)	39	39 (human)	
		427 (Fig. 5)	416	416 (human)	
		428 (Fig. 5)	417	417 (human)	
II	Keratocan (tetrapods)	9 (Fig. 6)	27	N.D.	
III	Mimecan (tetrapods)	13 (Fig. 7)	31	N.D.	
		26 (Fig. 7)	33		
III	Epiphycan (bony vertebrates)	16 (Fig. 8)	28	N.D.	
		27 (Fig. 8)	33		
		53 (Fig. 8)	46		
III	Opticin (bony vertebrates)	30 (Fig. 9)	N/A	N.D.	
		37 (Fig. 9)	56		
		46 (Fig. 9)	65		
		52 (Fig. 9)	71		

Prediction of tyrosine sulfation sites in the N- and C-terminal regions of human class II and III SLRPs based on the conservation of tyrosine residues and adjacent features promoting tyrosine sulfation**.** Tyrosines that remained after cut-off are listed with their positions in the MSA and sequence logo representations. The figure with the relevant MSA and logo is indicated in brackets. The corresponding tyrosine residues in the precursor human SLRP are listed and the residues, which are experimentally confirmed as sulfated, are indicated. Studies with identified tyrosine sulfations are cited

### The evolution of tyrosine-rich and acidic terminal extensions of class II and III SLRPs

**Fig. 4:**
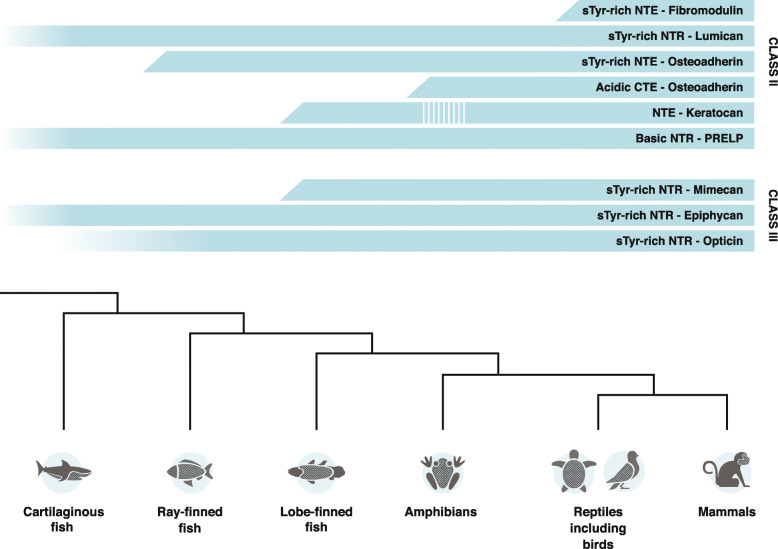
Emergence and development of major structural features in the N- and C-terminal regions of SLRPs. The major results of the study are illustrated. The upper panel illustrates the presence (cyan-bar) or absence (no bar) of elements in the variable N- and C-terminal regions of class II and III SLRPs, while the lower panel depicts the interrelationships of jawed vertebrates. Closed bars (cyan) represent that specific changes of the terminal region of a SLRP occurred between the splits of two lineages (see lower panel), thus pinpointing the evolutionary point of its development. Open faint bars represent the presence of a sulfotyrosine-rich or basic N-terminal region in all sequences in the dataset of that SLRP; hence, its development arose earlier in vertebrate evolution. No major changes occurred in the N-terminal region of lumican, PRELP, epiphycan or opticin during the evolution of jawed vertebrates (opticin-dataset does not contain sequences from cartilaginous fish). The sulfotyrosine-rich N-terminal extension of osteoadherin is the evolutionarily first observable change in the terminal regions of the SLRPs. The extension must have developed in a progenitor to all extant bony vertebrates since a sulfotyrosine-rich N-terminal region is not present in osteoadherin from cartilaginous fish but is present in all other sequences of the dataset. In the split between ray-finned and lobe-finned fish, an extension of the N-terminal region of keratocan developed in sarcopterygians (although it is not present in amphibians). Additionally, a sulfotyrosine-rich N-terminal region is generally present in mimecan of the sarcopterygian species. In the C-terminal region of osteoadherin, an extension developed containing a cluster of acidic residues after the split between lobe-finned fish and tetrapods. The evolutionarily newest structural feature in the terminal region of class II and III SLRPs happened in fibromodulin with the emergence of a sulfotyrosine-rich N-terminal extension in a progenitor to all extant amniotes. sTyr = sulfotyrosine; NTE = N-terminal extension; NTR = N-terminal region; CTE = C-terminal extension

The presence or absence of tyrosine-rich terminal regions in vertebrate class II and III SLRPs can theoretically be explained in this manner: The ancestor of extant jawed vertebrates had a single SLRP progenitor to all class I-III SLRPs without a tyrosine-rich N-terminal region, which then at later stages developed independently in the different vertebrate class II and III SLRPs through convergent evolution. The fact that SLRPa (an orthologues gene-product of the ancestral gene to all extant class I, II, and III SLRP genes [1]) of the sea squirt has very short terminal regions that are non-acidic and do not contain tyrosine clusters (Additional file 1**Fig. S11**) indicates that SLRPs of early chordates did not have tyrosine-rich terminal regions. This suggests that the tyrosine-rich terminal regions originated through convergent evolution at different points (Fig. 4). Sulfation-prone tyrosine-rich N-terminal regions are present in all sequences in the datasets of lumican, epiphycan and opticin and thus developed prior to the emergence of cartilaginous fish for lumican and epiphycan and prior to ray-finned fish for opticin. The same holds true for the proline-rich and basic N-terminal region of PRELP, which must have evolved in a progenitor to jawed vertebrates. Extensions of the osteoadherin N- and C-terminal regions occurred at different points in evolution. Thus, a cluster of sulfotyrosines in the N-terminal region of osteoadherin emerged in bony vertebrates after the divergence with cartilaginous fish, while after the split between lobe-finned fish and tetrapods, the acidic C-terminal extension of osteoadherin emerged in a progenitor to all extant tetrapods. After the split between ray-finned and lobe-finned fish, an N-terminal extension and a sulfotyrosine-rich N-terminal region developed in sarcopterygian keratocan and mimecan, respectively. Finally, a sulfotyrosine-rich N-terminal extension developed in amniote fibromodulin after the amphibian-amniote split (Fig. 4). Consequently, our results suggest that sulfation-prone tyrosine-rich N-termini of these class II and III SLRPs emerged and evolved through convergent evolution. Thus, in addition to several duplications of SLRPs in early chordates resulting in the emergence of a large array of functional SLRP proteins [1], selective pressures at different points during the evolution of vertebrate species resulted in the development of sulfotyrosine-rich and acidic N- and C-terminal regions in class II and III SLRPs. We believe the emergence and evolution of the N-terminal and C-terminal extensions of the SLRPs reflects altered functions of the proteins in connective tissues and thereby sheds light on vertebrate evolution. Sulfated and acidic terminal regions of SLRPs could be required to develop new lineage-specific, specialized extracellular matrices and tissues including endochondral bones, articular cartilage, and amnion and be used to seize and survive in new environments.

### Functional roles and proteolytic processing of (sulfo)tyrosine-rich and acidic terminal regions of class II SLRPs

Specific functional roles of the sulfotyrosine-rich N-terminal regions are only known for mammalian fibromodulin and are interactions with collagen and heparin-binding proteins including growth factors [25, 26]. The analysis of sequence data in our study reveals that these abilities of fibromodulin may have first developed in an ancestor to all extant amniotes since a tyrosine-rich N-terminal extension is absent in the other lineages of jawed vertebrates. The sulfotyrosine-rich N-terminal extension of fibromodulin provides the SLRP with an extra site for interactions with collagen [26]. This gave amniote fibromodulin the ability to interact with two tropocollagen molecules at once in addition to enhancing collagen fibrillogenesis and organization of the fibrils. Thus, the sulfotyrosine-rich N-terminal extension of fibromodulin could have had important roles in amniote tissue development. Interestingly, fibromodulin together with other SLRPs, is present in the amnion, a tissue unique to amniotes [27]. It is possible that the new functions enabled by the sulfotyrosine-rich extension were utilized by amniotes to develop a functioning amnion to protect embryos and foetuses during gestation or oviparity. Furthermore, as fibromodulin is essential for the scarless cutaneous wound healing in foetuses [28, 29], it is possible that the sulfotyrosine-rich N-terminal extension is involved in creating this phenotype by interacting with heparin-binding growth factors and regulating their signalling functions. For example TGFβ1, which is involved in scar formation and is a heparin-binding growth factor [29, 30], could be a potential interaction partner with the sulfotyrosine-rich N-terminal extension of fibromodulin.

In addition to these known functions, several studies suggest other roles for tyrosine-rich N-terminal regions in class II SLRP members. In diseased mammalian tissue, class II SLRPs can be cleaved or trimmed by proteases to various degrees. In an in vitro model for cartilage degradation, the N-terminal region of fibromodulin has been reported to be cleaved by MMP13 at Tyr63 to remove the sulfotyrosine-rich extension [31]. Interestingly, the sulfotyrosine-rich extension of fibromodulin has been found to bind MMP13 [25], and this could serve to sequester pro-MMPs to specific and relevant sites in the matrix until activation is needed. Additionally, in vitro cleavage by arthritis-associated ADAMTS-4 and -5 proteases occurs at the same site [32]. This cleavage must disturb the collagen binding of fibromodulin and its interactions with other binding partners, including the complement factor C1q which is known to contribute to the pathogenesis of arthritic diseases [25, 26, 33]. Fragmentation of fibromodulin with release of the sulfotyrosine-rich N-terminal region could therefore contribute to chronic arthritic disease development. Considering the involvement of fibromodulin in arthritic diseases and the emergence of the sulfotyrosine-rich N-terminal extension in the amniote clade, the selective pressure for this extension to evolve could have led to the development and homeostasis of cartilage that can withstand the wear and tear of movement in amniotes.

Lumican may affect tumour progression, and its sulfotyrosine-rich N-terminal region was shown to be cleaved off by specific MMPs associated with cancer cell invasion in one study [34]. The cleavage cancelled out the antitumour effect of lumican on a fibrosarcoma cell line. It was, however, not investigated whether this was directly due to the removal of the sulfotyrosine-rich N-terminus. Experimental evidence also suggests that lumican has a role in bacterial phagocytosis and protection against bacterial infections [35]. It was further shown that Tyr20 of lumican is important in this role. Our MSA reveals that Tyr20 is highly conserved and likely to be sulfated in all species containing a tyrosine at this position (Fig. 2b; position 3 and Table 1). This function could have driven selection of features favouring tyrosine sulfation in the N-terminal region of lumican.

In relation to osteoadherin’s roles in endochondral bone development and remodelling [36–40], it is tempting to suggest an involvement of its sulfotyrosine-rich N-terminal extension in these processes, as it is missing in the cartilaginous fishes whale shark and elephant shark. Further, the fact that these sharks are from the two different subclasses of cartilaginous fish supports the possibility that this tyrosine-rich N-terminal extension is missing in all cartilaginous fish. There could be several possible functions of the sulfotyrosine-rich N-terminal extension of osteoadherin in bones: 1) As it is highly negatively charged, it could serve to sequester calcium ions making them either available or unavailable for hydroxyapatite crystallization, which is a known function of other proteins [41]; 2) It could assist in controlling the diameter and shape of collagen fibrils to create a proper matrix for bone formation; or 3) It could act as a heparin-mimic providing osteoadherin the ability to interact with heparin-binding growth factors and other proteins involved in bone development or remodelling. In vitro and in vivo experiments focusing on the tyrosine-rich N-terminal region of osteoadherin could determine if this part of the protein is required for proper endochondral bone development. Osteoadherin has strong affinity for hydroxyapatite, and its C-terminal acidic extension has previously been suggested to mediate this interaction [39, 40]. Together with the observations from this study, this evidence suggests that the C-terminal extension is a tetrapod innovation (Fig. 2d **and** Fig. 4) for hydroxyapatite binding and regulation of mineral crystal growth. Thus, it is likely that both terminal extensions of osteoadherin are required for proper development of ossified endochondral bone in tetrapods.

Furthermore, the acidic and sulfotyrosine-rich N-terminal regions can act as heparin-mimics [25]; therefore, it is possible that a general role of this structural feature in the class II and III SLRPs is fine-tuning the regulation of heparin-binding growth factor stimuli, e.g., by sequestering them in the ECM until their actions are needed.

### Functional roles and proteolytic processing of (sulfo)tyrosine-rich N-terminal regions of class III SLRPs

Class III SLRPs can be found as proteolytically processed variants in different types of healthy mammalian tissues [8–10]. The proteolytic cleavages partly or fully removes the tyrosine-rich N-terminal regions. For example, in different mammalian tissues, mimecan is proteolytically processed into two distinct cleavage products, KSPG25 and osteoglycin, which both lack the tyrosine-rich N-terminal region and appear to have different functions [8]. For several proteins, proteolytic processing requires the presence of sulfotyrosines. For example, tyrosine sulfation of coagulation factors V and VIII is needed for their proper proteolytic activation by thrombin [42]. Hence, it is possible that the potential sulfotyrosine clusters of mimecan, epiphycan, and opticin interact with proteases and are required for their proteolytic processing. This modification could be required for proper functions of the SLRPs or for fine-tuning their functional roles upon specific stimuli. Specifically, the processing of mimecan into KSPG25 alters its regulation of collagen fibrillogenesis [43]. Additionally, the N-terminal fragments of class III SLRP members could relay signals to surrounding cells after their release, as observed with other protein fragments such as fragments from the class I SLRP biglycan [44].

### Conservation of an N-terminal glutamine (Q) residue provides proteolytic resistance to class II SLRPs

An N-terminal glutamine (Q) or glutamate (E) spontaneously, or through catalysis by glutaminyl cyclases, cyclizes into pyroglutamate (pQ) [45]. N-terminal pQ residues are known to protect mature extracellular proteins from trimming by aminopeptidases, thereby prolonging the protein half-life. Our MSA and sequence logo study reveals that an N-terminal Q is highly conserved in fibromodulin, especially in amniotes (Fig. 2a **and** Additional file 1**Fig. S2**). Thus, all fibromodulins containing an extended tyrosine-rich N-terminal region have an N-terminal Q. Additionally, an N-terminal Q is also found in most lumican sequences (Fig. 2b **and** Additional file 1**Fig. S3**) and is completely conserved in all retrieved PRELP sequences (Fig. 2f **and** Additional file 1**Fig. S7**) and in all osteoadherin sequences with the exception of Ghost shark which does not have an extended tyrosine-rich N-terminal (Fig. 2c **and** Additional file 1**Fig. S4**). In contrast to the other class II SLRPs, keratocan appears to lack a conserved N-terminal Q or E (Fig. 2e **and** Additional file 1**Fig. S6**), suggesting that degradation of its N-terminal region by aminopeptidases is less critical for its functional role. Notably, according to the MSA and sequence logo analyses, mature mimecan, epiphycan and opticin do not have N-terminal Q or E residues (Fig. 1). Thus, our results suggest that the class III SLRPs are less sensitive to degradation of their N-terminal region by aminopeptidases compared to the class II SLRPs; alternatively, it is possible that the N-terminal regions of class III SLRPs are removed through natural processing, and there has not been the same selection pressure to preserve their tyrosine-rich N-terminal regions. Altogether, our study indicates that an N-terminal Q in mature class II SLRPs is evolutionary conserved to protect the sulfotyrosine-rich N-terminal regions (or proline and arginine-rich N-terminal region in the case of PRELP) against non-specific exoproteolytic trimming and thereby shields their functional roles.

## Conclusion

The multiple sequence analyses show that tyrosine-rich N-terminal extensions with consensus features promoting tyrosine sulfation developed at different points in the evolution of class II and III SLRPs. Using the conservation of tyrosines and adjacent residues, we present a novel approach for predicting tyrosine sulfation sites in the N- and C-terminal regions of class II and III SLRPs. We propose that this tyrosine sulfation prediction approach can also be applied to other sulfotyrosine-containing proteins. The emergence of sulfotyrosine-rich N-terminal extensions in some class II and III SLRPs suggests that these post-translational modifications have provided the SLRPs with unique functions that have been vital in developing new specialized tissues during the evolution of vertebrate species.

## Methods

### Sequence collection

A minimum of three sequences annotated as the SLRPs of interest were retrieved from the UniProtKB database (Swiss-Prot and TrEMBL, release 2017_03) for each taxonomic class level. Sequences for some taxonomic classes were retrieved from the National Center for Biotechnology Information (NCBI) to achieve a minimum of three sequences when possible. The sequences were assigned to datasets labelled with their SLRP name. All sequences retrieved belonged to jawed vertebrates. Efforts to identify class II and III SLRP sequences from jawless fish (*agnatha*) and cephalochordates (*cephalochordata*) through sequence similarity searches (BLAST, NCBI database [46]) were unsuccessful.

### Sequence assessment and annotation

Manual assessment of the sequences was conducted as a considerable number of the retrieved sequences contained problematic features as described below. The manual assessment was done to ensure that the data were of sufficiently high quality to achieve optimal alignments revealing the evolution of SLRPs. Prerequisites for the sequences to be added to the datasets were: 1) Sequences annotated as another protein were excluded; 2) A canonical N-terminal cysteine cluster motif and C-terminal conserved cysteines were required; 3) Fragmented sequences were excluded; 4) Sequences without annotated signal peptides or with a non-methionine residue at the N-terminus were excluded; 5) Sequences with unknown residues (X) were excluded; and 6) Only one sequence of the same gene product from the same species was retained in the dataset. For the ray-finned fish epiphycan, the short splice variants were chosen for this study to better correspond to the other taxonomic classes. The long splice variants of the ray-finned fish epiphycan, as described by W. Zhou et al. [47], have therefore been excluded from the multiple sequence alignments.

### Phylogenetic relationships and dendrograms

Multiple sequence alignments (MSAs) of each class of SLRPs, generated using CLUSTAL OMEGA v1.2.4 with the default settings [48], were used to construct phylogenetic trees to confirm that the remaining sequences were correctly annotated and categorized in the right SLRP dataset. MEGA7.0.26 [49] was used to construct phylogenetic trees by the maximum likelihood method based on all sites in the alignments including gaps. The phylogeny was tested using 1000 Bootstrap replications. The final datasets include a total of 515 sequences of biochemically characterized SLRPs and predicted SLRPs deduced from transcriptional and genomic sequence data from jawed vertebrates (*Gnathostomata*).

The introductory dendrogram was created from Human SLRP sequences retrieved from the Swiss-Prot database and aligned by the ClustalW 2.0.12 algorithm through Jalview 2.10.1. The phylogenetic tree based on the aligned sequences was constructed by Neighbour-joining with deletion of gaps using the MEGA7.0.25 suite. iTOL v3 was used to present the phylogenetic tree.

### Sequence analysis and representation

The M-Coffee (V11.00.d625267) software only utilizing Mt_coffee_msa and Mmafft_msa in the alignment computation library was used to combine the advantages of the two methods, namely, T-Coffee’s general high accuracy with MAFFT’s advantage in aligning sequences with N- and C-terminal extensions [50, 51]. MSAs for each SLRP (Additional file 1**Figs. S2-S10**) provided the basis for presenting alignments with representative sequences of SLRP N- and C-terminal regions (Figs. 2 and 3). The N-terminal regions of SLRPs are defined as the sequences from the first residue after the signal peptide to the first cysteine residue in the conserved cysteine cluster motif. The C-terminal region of osteoadherin is defined as the sequence following the last conserved cysteine residue. The MSAs are represented by using Jalview 2.10.4 software [52]. The complete MSAs (with signal peptides removed) can be downloaded as Additional files 2 (Fibromodulin), 3 (Lumican), 4 (Osteoadherin), 5 (Keratocan), 6 (PRELP), 7 (Mimecan), 8 (Epiphycan), and 9 (Opticin), and can be accessed using common MSA workbenches such as Jalview.

Sequence logos were created using WebLogo 3.6.0 with the “equiprobable composition” setting [53]. The sequences used to create the logos were from specific taxonomic classes, as noted in the figure legends. The properties of amino acids in relation to tyrosine sulfation are indicated with colours as follows: Green = Y; Red = Acidic residues (E and D); Orange = Turn-inducing residues (P, G, N and S); Blue = Basic and hydrophobic residues (R, K, H, F, W, I, M, V and L); Pink = C; and Black = Neither induces nor inhibits tyrosine sulfation (Q, A, and T). This colour scheme is also used in the MSAs.

### Tyrosine sulfation prediction

Sulfotyrosine prediction software was not used as it has previously been shown to be inaccurate in identifying experimentally proven sulfotyrosine sites in SLRPs [22]. A tyrosine sulfation prediction was therefore performed based on evolutionary conservation of tyrosine sites and by the features promoting or suppressing tyrosine sulfation (Table 2). Three cut-off values were selected for the prediction of sulfated tyrosines based on the bioinformatics data in this study. The cut-off values are: 1) A minimum of 75% tyrosines at a specific position (75% consensus value); 2) The ratio between residues promoting and supressing tyrosine sulfation within ±5 positions of the tyrosine should be 50% or higher, and the type of residue is the most frequent residue at that position (the tallest letter in the sequence logo representation); and 3) Gaps need to have a frequency of less than 50% at the positions being analysed (occupancy value of at least 50%).

**Table 2:** Common features in the proximity of tyrosine sulfation sites

Feature	Position from Tyr (Y)	Description
Prevalence of acidic residues	±5	Presence of several Glu (E) or Asp (D) residues near the Tyr (Y). Frequently at the − 1 position.
Prevalence of turn-inducing residues	±7	Several Pro (P), Gly (G), Asn (N), Asp (D) or Ser (S) residues present within seven residues of the Tyr (Y).
Limited basic and hydrophobic residues	±5	Only few Arg (R), Lys (K), His (H), Phe (F), Trp (W), Ile (I), Met (M), Val (V) or Leu (L) residues near the Tyr (Y).
Absence of disulfide bonds	±7	No disulfide-bonded Cys (C) close to the Tyr (Y).
Absence of N-linked glycosylation sites	N/A	No N-linked glycosylation consensus sequence (N-X-S/T) in vicinity of the Tyr (Y).
Prevalence of several tyrosine sulfation sites	N/A	Tyr (Y) residues within a tyrosine cluster containing features promoting tyrosine sulfation.

Amino acid residues and modifications that are absent or commonly found in proximity to experimentally determined sulfotyrosines

## Supplementary information

**Additional file 1.** Supplementary Figures S1-S11 and Supplementary Tables S1-S3. PDF format file (.pdf) of phylogenetic trees of class II and III SLRPs (Fig. S1), N- or C-terminal regions of the complete multiple sequence alignments for each SLRP dataset (Fig. S2-S10), SLRPa sequence from *Ciona intestinalis* (Fig. S11), and tables with number of residues and tyrosines or arginines/lysines in the N- or C-terminal regions of class II and III SLRPs (Tables S1-S3).

**Additional file 2.** Fibromodulin. FASTA format file (.fasta) containing the complete multiple sequence alignment of the fibromodulin dataset with signal peptides removed. May be accessed using common multiple sequence alignment workbenches such as Jalview.

**Additional file 3.**  Lumican. FASTA format file (.fasta) containing the complete multiple sequence alignment of the lumican dataset with signal peptides removed. May be accessed using common multiple sequence alignment workbenches such as Jalview.

**Additional file 4.** Osteoadherin. FASTA format file (.fasta) containing the complete multiple sequence alignment of the osteoadherin dataset with signal peptides removed. May be accessed using common multiple sequence alignment workbenches such as Jalview.

**Additional file 5.**  Keratocan. FASTA format file (.fasta) containing the complete multiple sequence alignment of the keratocan dataset with signal peptides removed. May be accessed using common multiple sequence alignment workbenches such as Jalview.

**Additional file 6.** PRELP. FASTA format file (.fasta) containing the complete multiple sequence alignment of the PRELP dataset with signal peptides removed. May be accessed using common multiple sequence alignment workbenches such as Jalview.

**Additional file 7.** Mimecan. FASTA format file (.fasta) containing the complete multiple sequence alignment of the mimecan dataset with signal peptides removed. May be accessed using common multiple sequence alignment workbenches such as Jalview.

**Additional file 8.** Epiphycan. FASTA format file (.fasta) containing the complete multiple sequence alignment of the epiphycan dataset with signal peptides removed. May be accessed using common multiple sequence alignment workbenches such as Jalview.

**Additional file 9.** Opticin. FASTA format file (.fasta) containing the complete multiple sequence alignment of the opticin dataset with signal peptides removed. May be accessed using common multiple sequence alignment workbenches such as Jalview.

## Data Availability

Data from this study is available in Additional files 1-9.

## Abbreviations

GAG: Glycosaminoglycan
MSA: Multiple sequence alignment
PRELP: Proline-arginine-rich end leucine-rich repeat protein
PTM: Post-translational modification
SLRP: Small leucine-rich repeat protein
