# Supplementary material for: The origins and developments of sulfation-prone tyrosine-rich and acidic N- and C-terminal extensions of class ll and lll small leucine-rich repeat proteins shed light on connective tissue evolution in vertebrates
Source: BMC Evol Biol. 2020 Jun 23;20:73. doi: 10.1186/s12862-020-01634-3 (PMC7310474; doi:10.1186/s12862-020-01634-3)
Supplement: Supplementary file 1 — Additional file 1. Supplementary Figures S1-S11 and Supplementary Tables S1-S3. PDF format file (.pdf) of phylogenetic trees of class II and III SLRPs (Fig. S1), N- or C-terminal regions of the complete multiple sequence alignments for each SLRP dataset (Fig. S2-S10), SLRPa sequence from Ciona intestinalis (Fig. S11), and tables with number of residues and tyrosines or arginines/lysines in the N- or C-terminal regions of class II and III SLRPs (Tables S1-S3). [file 12862_2020_1634_MOESM1_ESM.pdf]

# Supplementary Information

## The Origins and Developments of Sulfation-prone Tyrosine-rich and Acidic N- and C-terminal Extensions of Class ll and lll Small Leucine-rich Repeat Proteins Shed Light on Connective Tissue Evolution in Vertebrates

Morten M. Jensen[1] & Henrik Karring[1]*

Affiliations: [1]Department of Chemical Engineering, Biotechnology and Environmental Technology, University of Southern Denmark, Campusvej 55, 5230 Odense, Denmark

*Corresponding author: Email: hka@kbm.sdu.dk

**A**

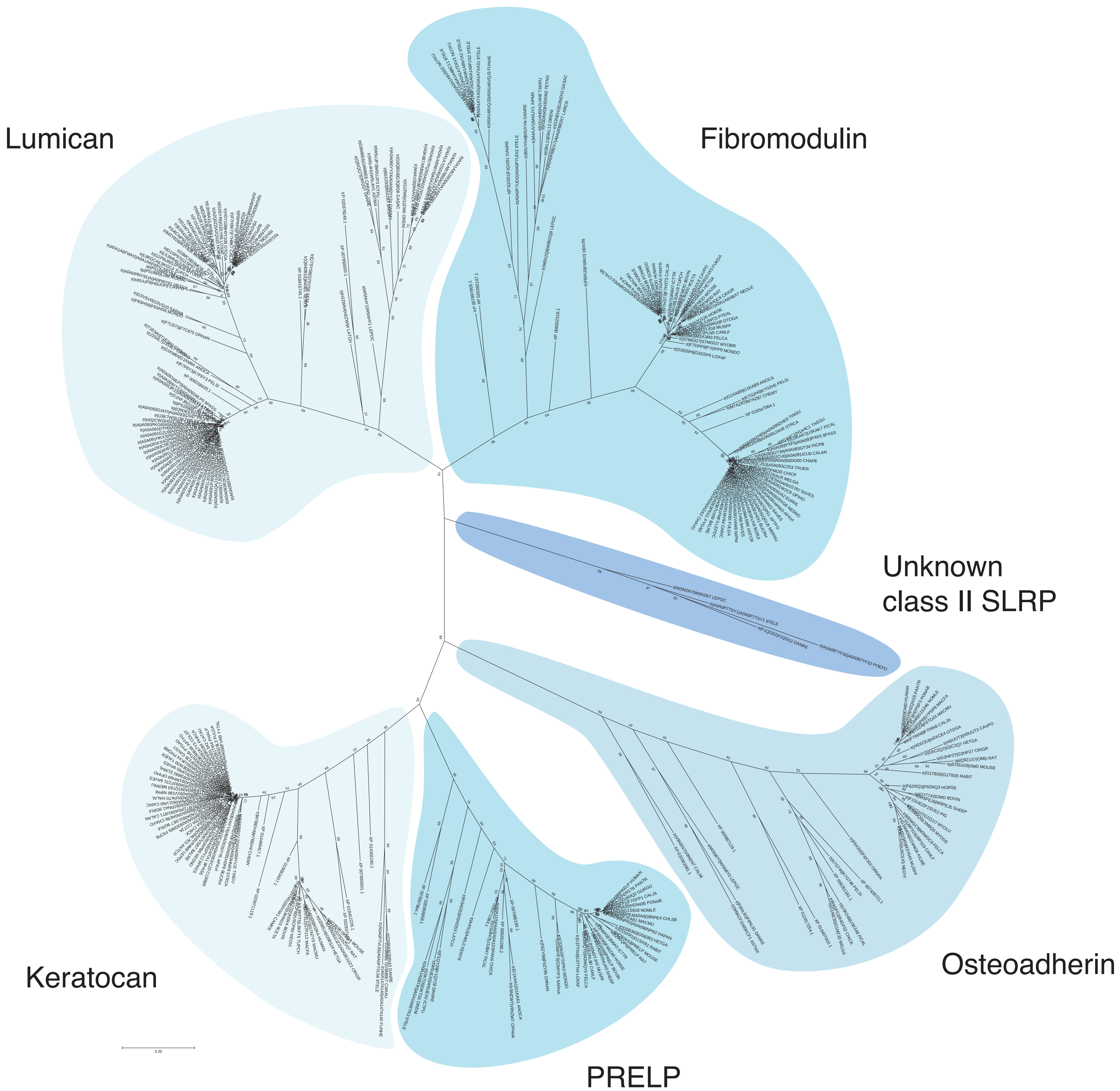
Lumican
Fibromodulin
Unknown class II SLRP
Osteoadherin
PRELP
Keratocan

**B**

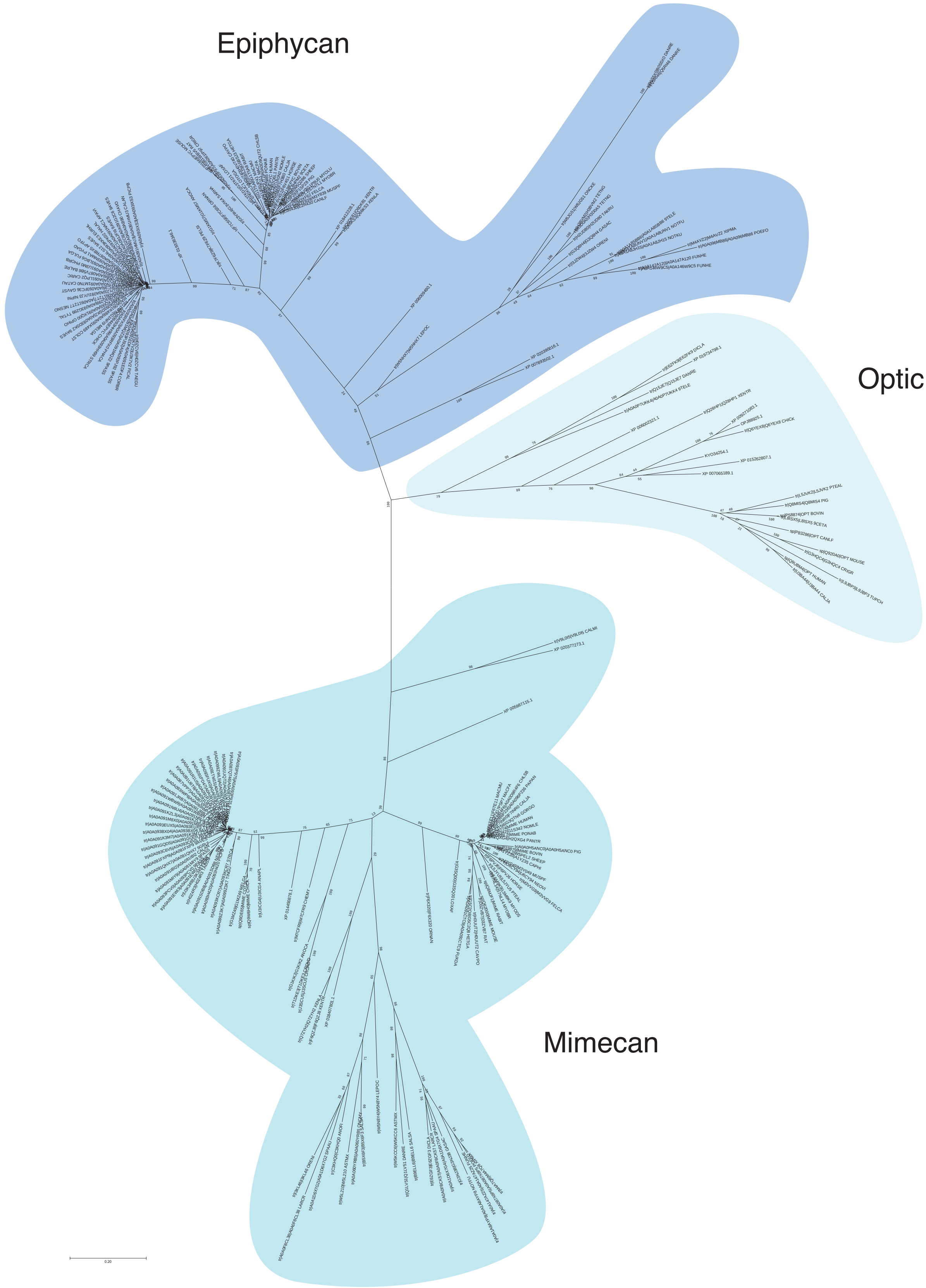

**Figure S1: Separate phylogenetic trees of class II and class III SLRPs.** Zoom in to see details. Phylogenetic trees are presented as radiated unrooted trees. The trees are drawn to scale with branch lengths measured in the number of substitutions per site (scale bar corresponds to 0.20 substitutions per site). The values on the nodes are bootstrap percentage values. Zoom in to view details. **A:** Phylogenetic tree of class II SLRPs. The sequences organized into six groups: one for each of the five class II SLRPs and one containing four sequences denoted as "unknown class II SLRP". These four sequences were annotated as lumicans. **B:** Phylogenetic tree of class III SLRPs. The sequences from the datasets of class III SLRPs arranged into three groups; one for each class III SLRP. The sequence ">tr|A0A0P7UKK4|A0A0P7UKK4_9TELE" rooted together with opticin sequences, although it is annotated as an epiphycan.

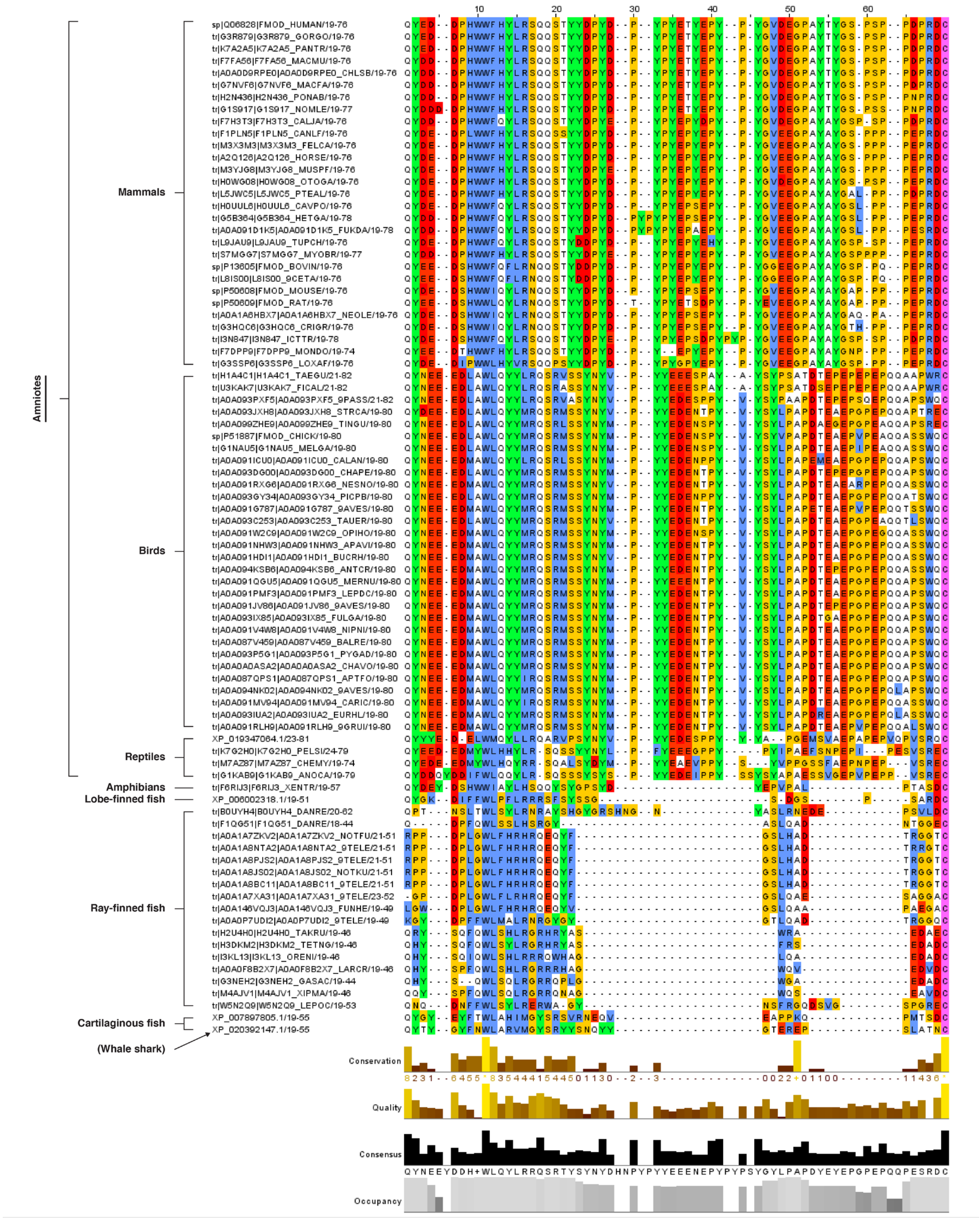


**Figure S2: A tyrosine-rich extension of the N-terminal region of fibromodulin is only present in amniotes.** Zoom in to see details. The N-terminal region of all aligned sequences in the fibromodulin dataset (84 sequences). An extended internal tyrosine-rich sequence of the N-terminal region of fibromodulin is evident in amniotes (position 30-64). This extension is also rich in amino acids promoting tyrosine sulfation. Hence, the N-terminal regions of anamniotes are shorter, and contain fewer tyrosines and features favouring sulfation of tyrosines. The N-terminal regions are relatively conserved within classes.

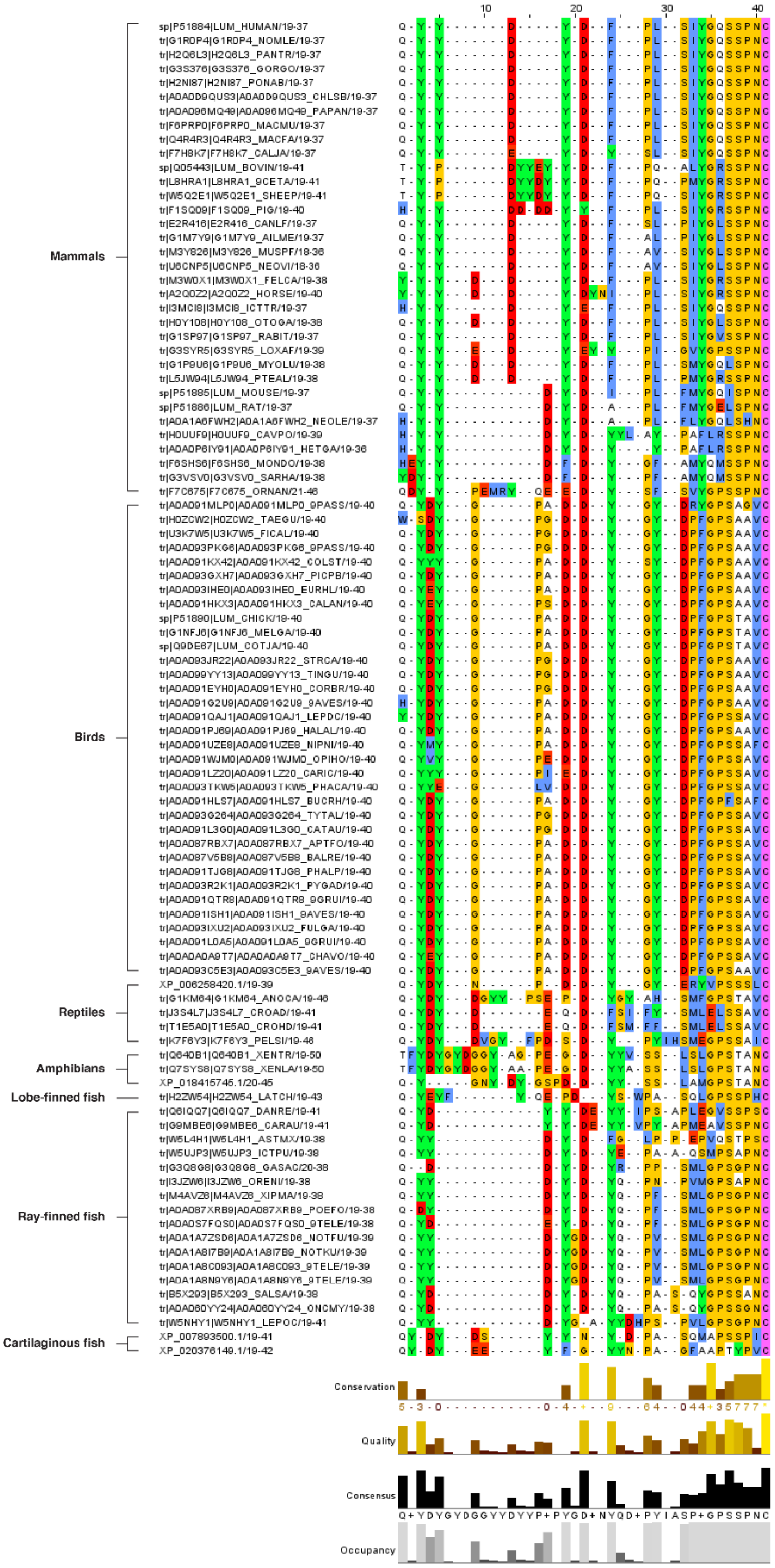


**Figure S3: A tyrosine-rich N-terminal region with features promoting tyrosine sulfation is found in lumican of all jawed vertebrates**. Zoom in to see details. The N-terminal region of all aligned sequences in the lumican dataset (95 sequences). All sequences in the dataset contain tyrosines together with features favouring sulfation. However, small variations can be observed such as more tyrosines in even-toed ungulates (*Artiodactyla*) and a small extension containing tyrosines and sulfation-promoting amino acids in the *Xenopus* genus.

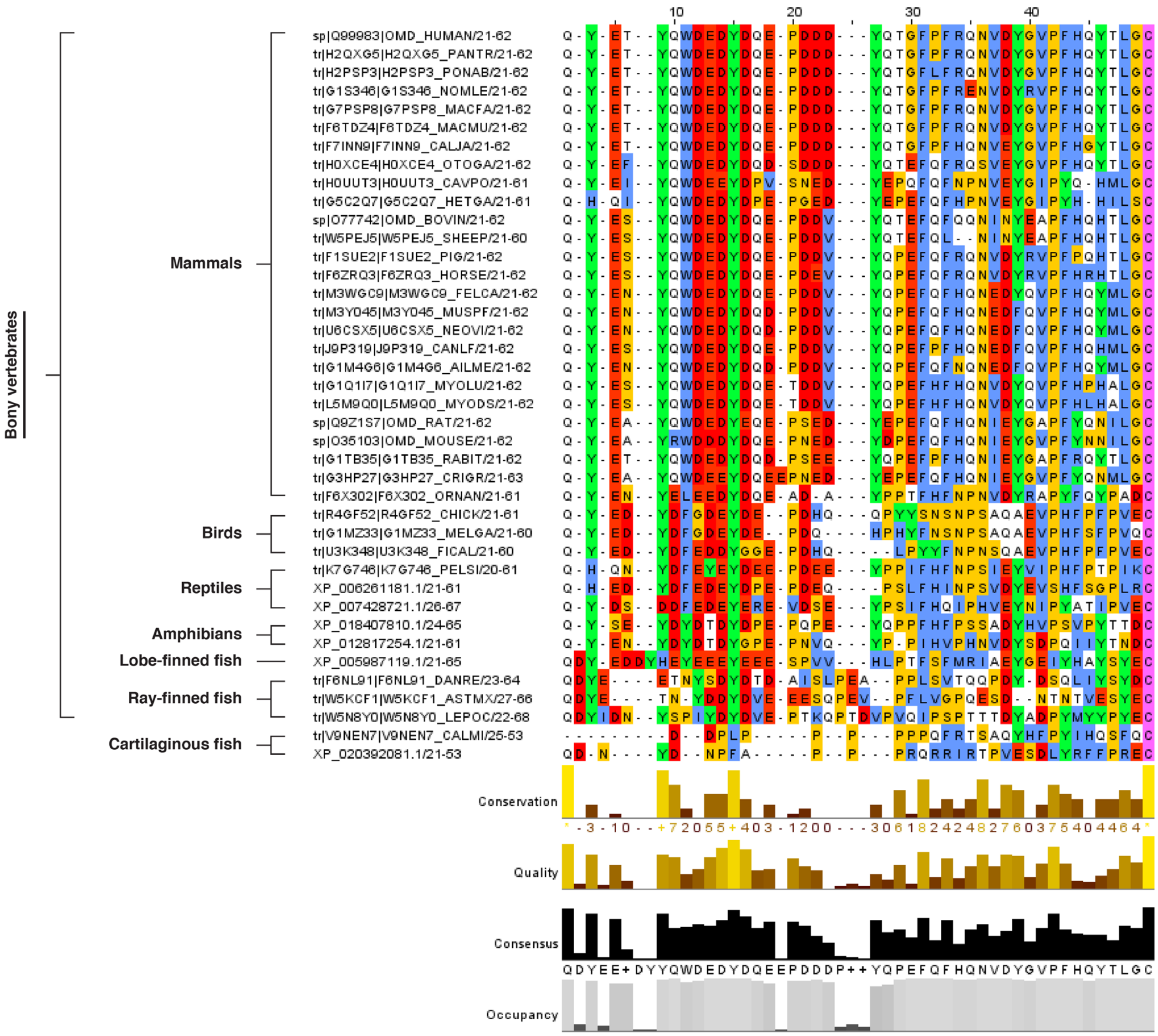


**Figure S4: An acidic and tyrosine-rich N-terminal extension is observed in osteoadherin from bony vertebrates, while it is not present in cartilaginous fish.** Zoom in to see details. The N-terminal region of all aligned sequences in the osteoadherin dataset (40 sequences). Tyrosines can be found together with a large degree of acidic residues in approximately the first half of the N-terminal region of osteoadherin from bony vertebrates. These features are not found in cartilaginous fish, and the N-terminal region is consequently shorter. An N-terminal glutamine is found in all sequences with the exception of ghost shark (V9NEN7_CALMI) osteoadherin.

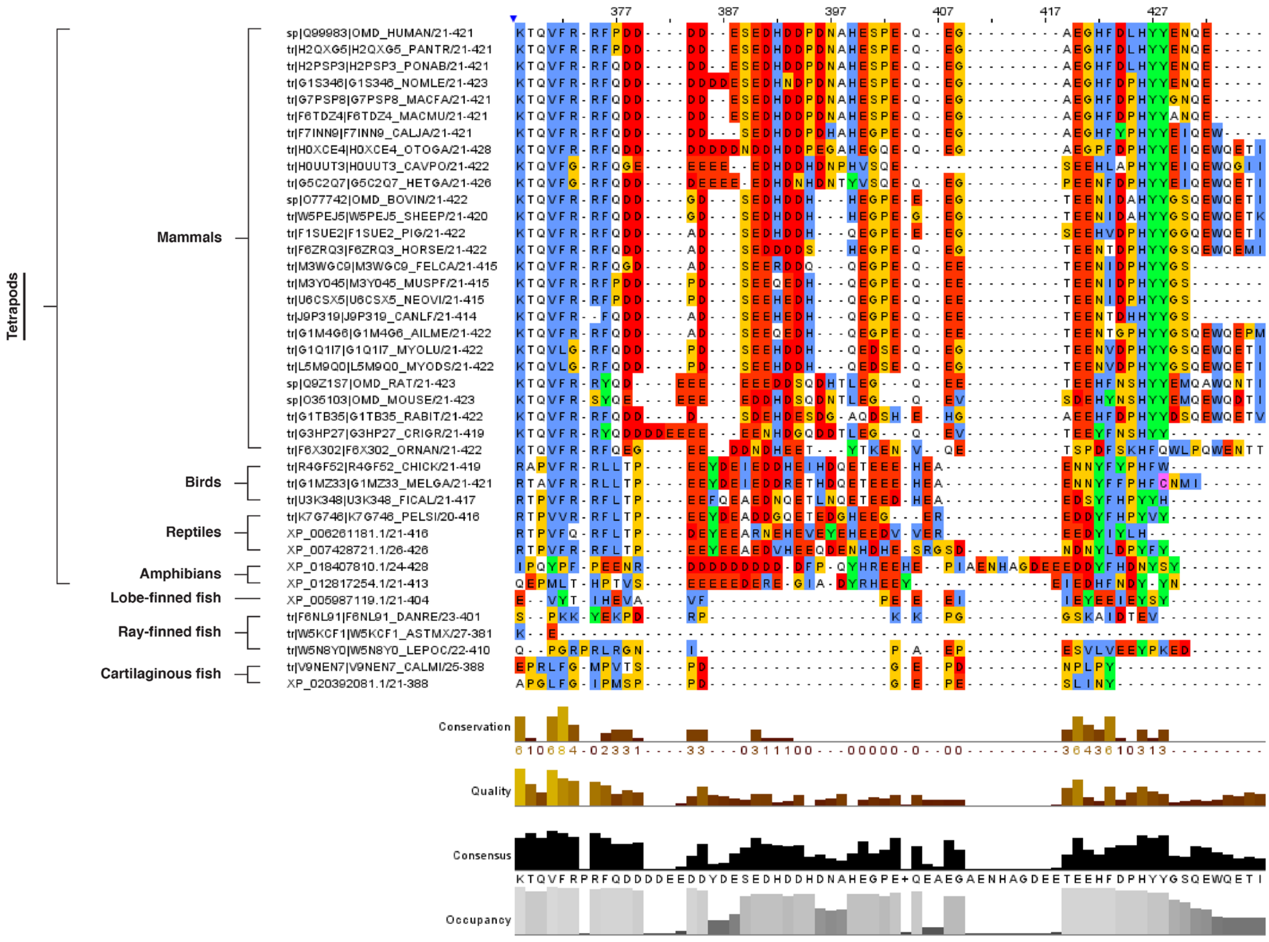


**Figure S5: Tyrosines with sulfation-promoting residues are present in all jawed vertebrates, while an acidic extension is only present in tetrapods.** Zoom in to see details. The C-terminal region of all aligned sequences in the osteoadherin dataset (40 sequences). The MSA reveals that tyrosines with some neighbouring residues that promote sulfation are present in all sequences. An acidic region of osteoadherin (position 384-409) is only present in tetrapods. Some features are unique to a single sequence, e.g., *N. parkeri´s* (XP_018407810.1) extra extended acidic region (positions 410-418) and *A. mexicanus´s* (W5KCF1_ASTMX) short C-terminal region (ending at position 371).

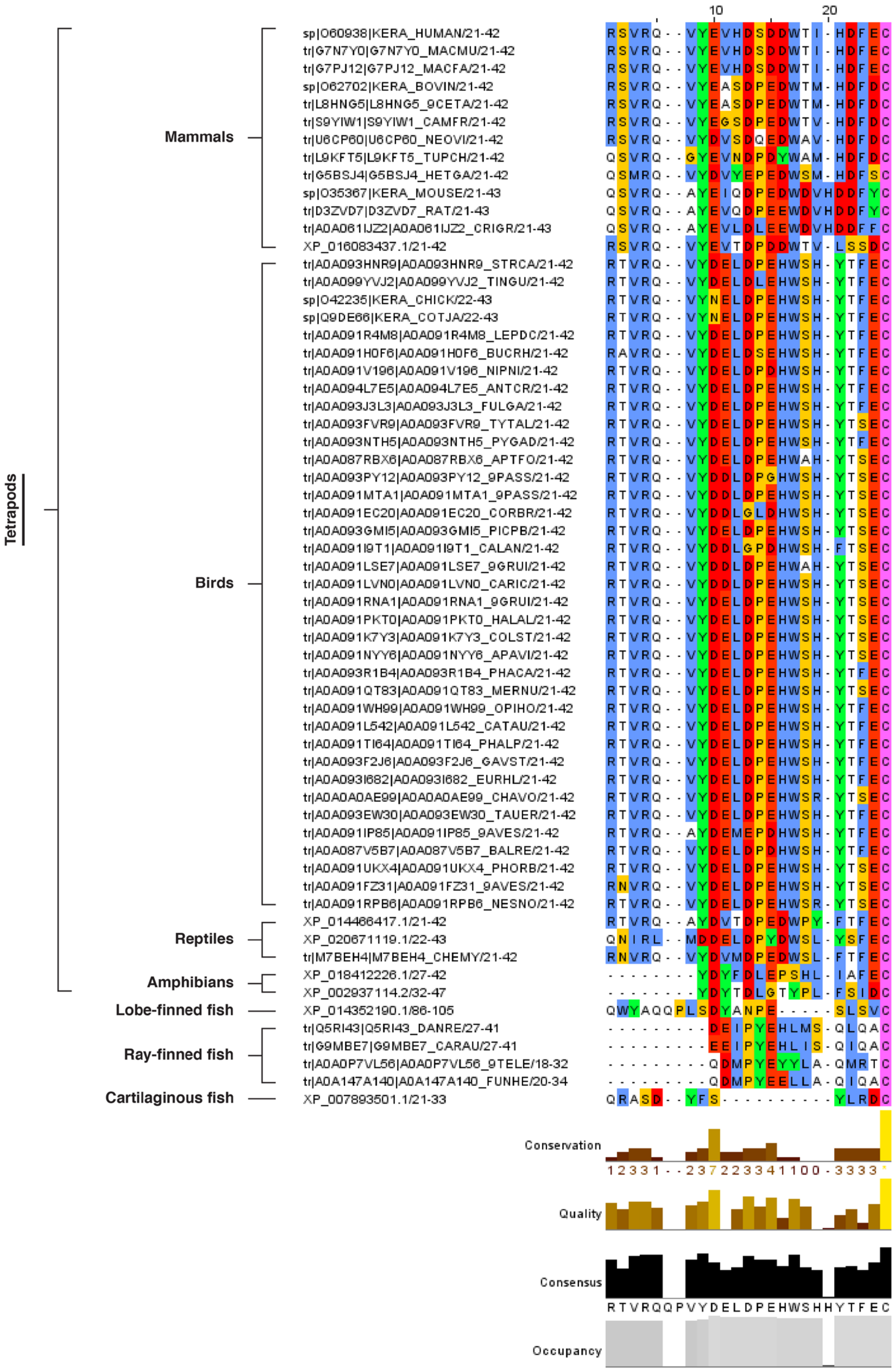


**Figure S6: All keratocan sequences contain at least one tyrosine together with sulfation-favouring amino acids.** Zoom in to see details. The N-terminal region of all aligned sequences in the keratocan dataset (61 sequences). One to three tyrosines can be observed in the N-terminal region of the sequences in the keratocan dataset. Tyrosine at position 9 is present in all sequences from tetrapods with the exception of the central bearded dragon (XP_020671119.1). Amino acids promoting sulfation are located C-terminal to the tyrosine at position 9 in tetrapods. Tyrosines with sulfation-promoting amino acids are also present in the coelacanth (XP_014352190.1), ray-finned fish and ghost shark (XP_007893501.1) keratocan sequences. The N-terminal regions of ghost shark (XP_007893501.1), ray-finned fish and the two sequences of amphibian opticin are shorter (as evident from the introduced gaps) than the N-terminal regions of amniote and coelacanth (XP_014352190.1) keratocan.

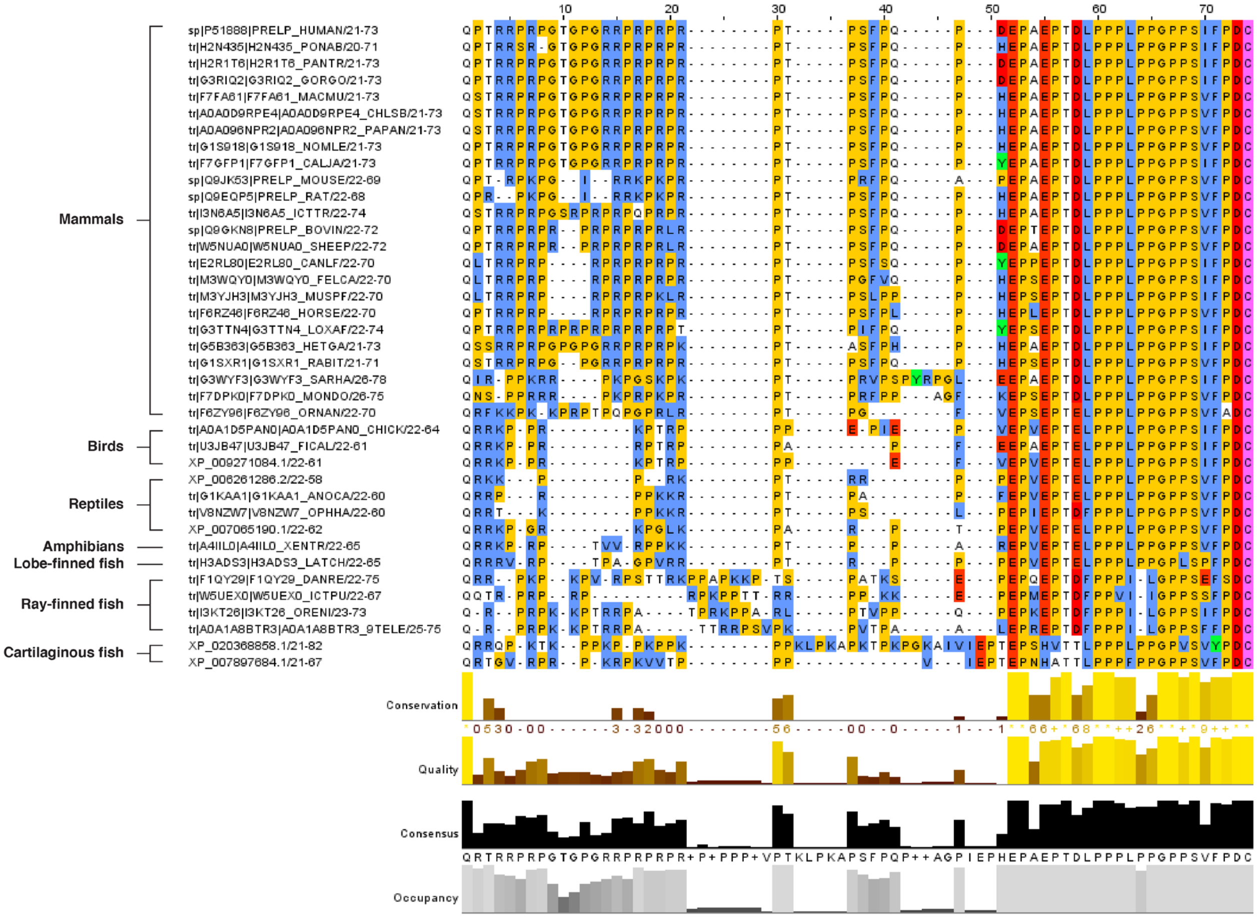


**Figure S7: All PRELP sequences contain a proline/basic residue-rich N-terminal region**. Zoom in to see details. The N-terminal region of all aligned sequences in the PRELP dataset (39 sequences). The last part of the N-terminal region (positions 51-73) is quite conserved, while a larger variation can be observed in the first part (positions 1-50) between the sequences. There is also variation in the ratio between arginine and lysine residues; e.g., human PRELP (P51888) only contains arginines while whale shark PRELP (XP_020368858.1) contains two arginines and ten lysines. All sequences of the dataset contain an N-terminal glutamine.

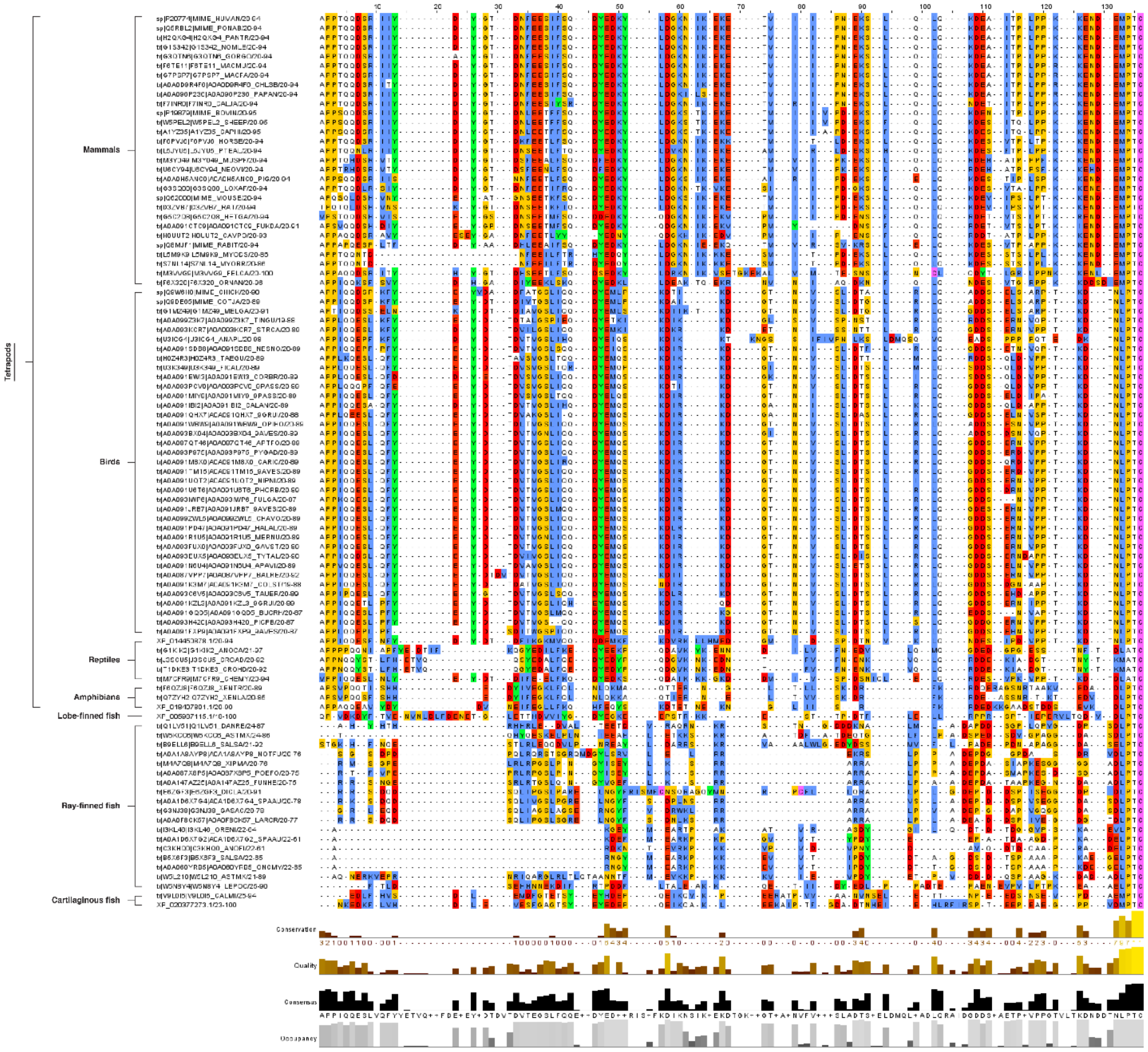


**Figure S8: The highly variable N-terminal region of mimecan contains tyrosines with concomitant sulfation-promoting residues**. Zoom in to see details. The N-terminal region of all aligned sequences in the mimecan dataset (95 sequences). It can be observed from the numerous gaps introduced into the MSA that the N-terminal region of mimecan is highly variable between jawed vertebrate species. Based on the number of tyrosines and their neighbouring residues, it appear that a cluster of sulfotyrosines is possible in the N-terminal region of all tetrapod mimecans with the exception of few species which only contain one or two tyrosines in the N-terminal region. Ray-finned and cartilaginous fish only contain 1-2 tyrosines in the N-terminal region of their mimecans, and a cluster of sulfotyrosines is therefore not possible. However, for some species, tyrosine sulfation of one or two residues still appears feasible.

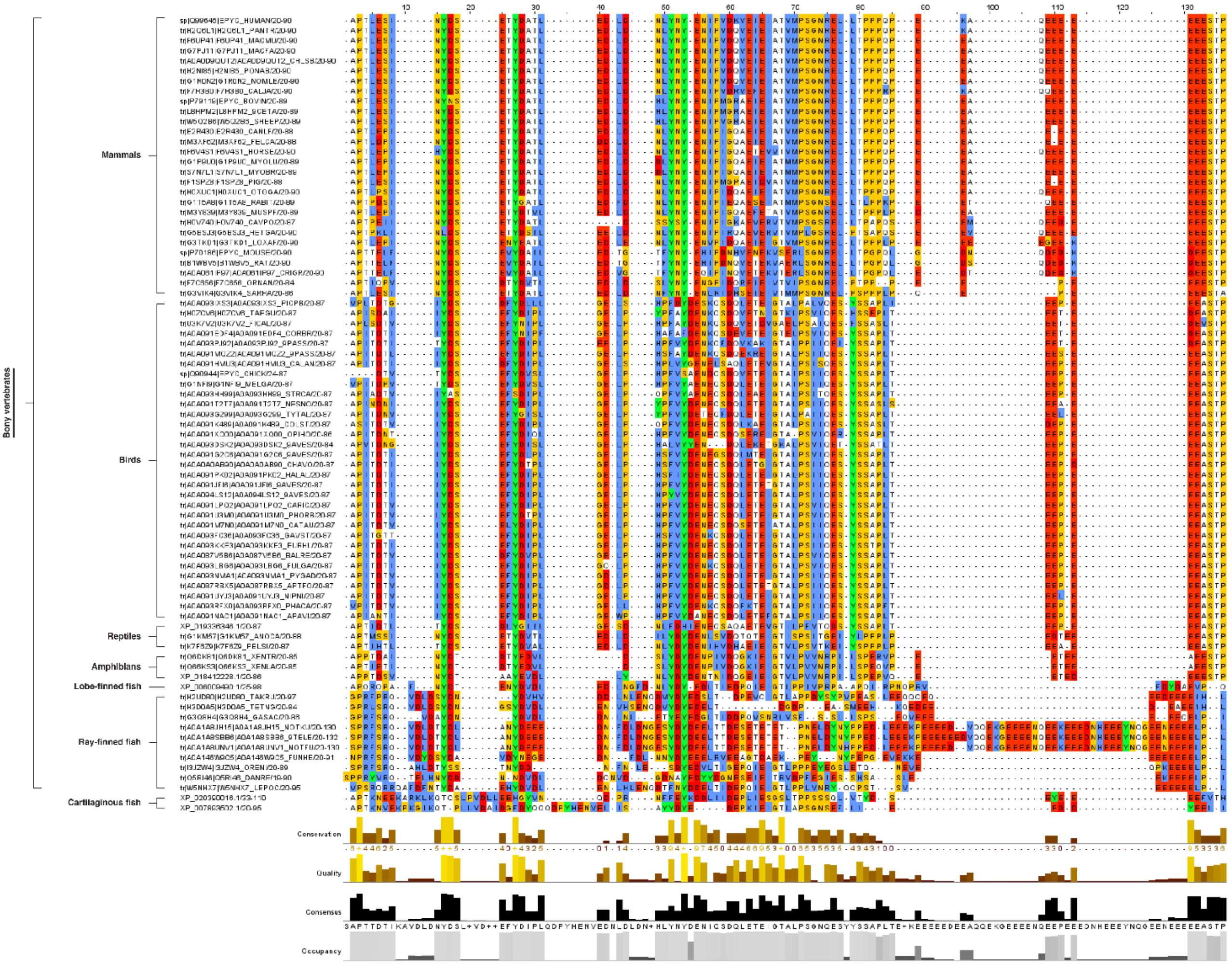


**Figure S9: All sequences of epiphycan from jawed vertebrates contain tyrosines with adjacent sulfation-promoting residues.** Zoom in to see details. The N-terminal region of all aligned sequences in the epiphycan dataset (79 sequences). The figure only shows from residues 1-90 (in humans) due to the long length of the N-terminal region in the alignment. All epiphycan sequences in the dataset contain tyrosines in the N-terminal region with adjacent residues promoting tyrosine sulfation. Inserts can be observed in the N-terminal regions of epiphycans from some animals; e.g., a few ray-finned fish have inserts from positions 92-124, while ray-finned fish and cartilaginous fish have different inserts at positions 9-48.

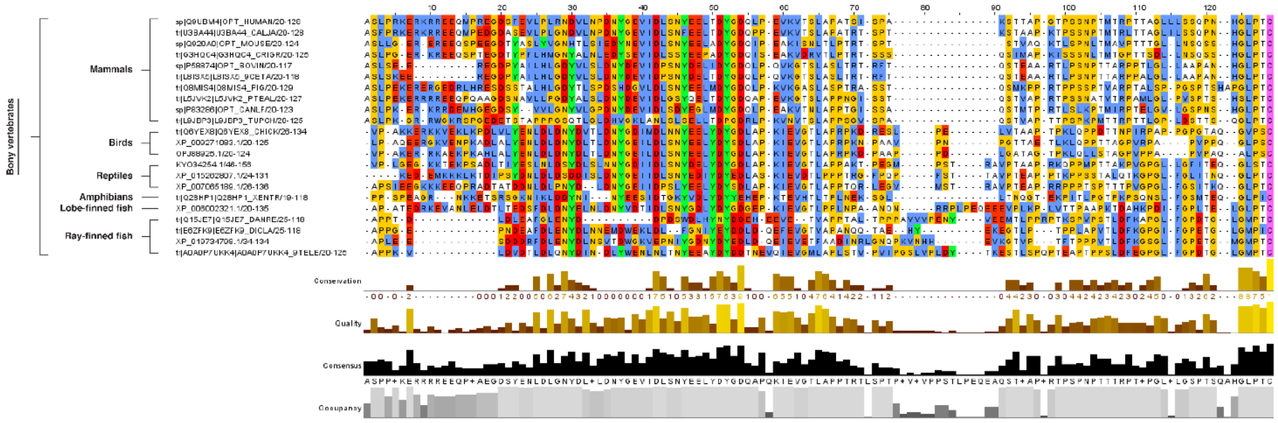


**Figure S10: A cluster of tyrosines with amino acids favouring sulfation is conserved in the N-terminal of opticin in bony vertebrates.** Zoom in to see details. The N-terminal region of all aligned sequences in the opticin dataset (22 sequences). All opticin sequences in the dataset contain tyrosines in the N-terminal region, with the exception of the Chinese tree shrew (L9JBP3_TUPCH). The tyrosines are mainly clustered in the first half of the N-terminal region together with other amino acids promoting tyrosine sulfation. Some variation between the sequences may be observed such as internal stretches of amino acids that are missing in the *Bos* genus and in ray-finned fish.

>tr|F6TTX9|F6TTX9_CIOIN decorin-like OS=Ciona intestinalis OX=7719 GN=LOC100178772 PE=4 SV=2

MRFFSQVTLFLCIAVLCTSPTTFGSRFKRGSRRQPVLFNRYICPPQCACSLNIIYCSGKQLNEIPSTFPRNGEFLHLENN
YITRIHSGVFRHFPAIQRIILTKNRLISAGLKARSFEGLTALKRLDLSENKLTRFPRSLPPSLVELRLNLNNITKVKRGA
TRGLTNLVALSLFRNSITDAGFEPAILKNMTALSYLDLNENLLETVPQGLPESLREIRLENNGLKNVTAGIFTSQSLLHH
FSLRNNQLSDQGINFNAWSNMSNLFLLDLSYNKLRTIPRGLPPSLHQLIIENNFIEEINYKTFISSLNLTQIKLSFNKIR
IITPGSFTRLIHLRFLDLAFNNLLYVPRGLPTTLEALFLESNQIFSVQIDSFCPMNPERFVTSKLHTLRLDGNLIDSNDL
PALAFYCLSSMEVVVT

**Figure S11: SLRPa sequence from *Ciona intestinalis.*** SLRPa is an orthologue of the ancestral gene product to all extant class I, II and III SLRPs. Grey = Signal peptide; Blue = N-terminal region; Purple = Leucine-rich repeat domain; Red = C-terminal region.

**Table S1: Number of residues and tyrosines in the N-terminal region of the class II SLRPs fibromodulin, lumican, osteoadherin, keratocan and in the C-terminal region of osteoadherin at the taxonomic class level.**

| Taxonomic class | Number of residues in the N-terminal region | Number of tyrosines in the N-terminal region |
|---|---|---|
| | **Fibromodulin** | |
| Mammals | 55-59 | 10-12 |
| Birds | 61 | 10 |
| Reptiles | 55-60 | 9-11 |
| Amphibians | 38 | 7 |
| Lobe-finned fish | 32 | 2 |
| Ray-finned fish | 25-42 | 1-4 |
| Cartilaginous fish | 36 | 4-8 |
| | **Lumican** | |
| Mammals | 18-25 | 4-6 |
| Birds | 21 | 3-5 |
| Reptiles | 20-27 | 2-6 |
| Amphibians | 25-31 | 5-6 |
| Lobe-finned fish | 24 | 4 |
| Ray-finned fish | 18-22 | 2-6 |
| Cartilaginous fish | 22-23 | 5-6 |
| | **Osteoadherin (N-terminal)** | |
| Mammals | 39-41 | 4-7 |
| Birds | 39-40 | 4-5 |
| Reptiles | 40-41 | 3-5 |
| Amphibians | 40-41 | 7 |
| Lobe-finned fish | 44 | 8 |
| Ray-finned fish | 39-46 | 4-9 |
| Cartilaginous fish | 28-32 | 2 |
| | **Osteoadherin (C-terminal)** | |
| Mammals | 65-75 | 3-6 |
| Birds | 66-69 | 4-5 |
| Reptiles | 64-68 | 6 |
| Amphibians | 63-76 | 6-7 |
| Lobe-finned fish | 48 | 6 |
| Ray-finned fish | 24-51 | 2-3 |
| Cartilaginous fish | 44 | 1-2 |
| | **Keratocan** | |
| Mammals | 21-22 | 1-2 |
| Birds | 21 | 1-2 |
| Reptiles | 21 | 1-2 |
| Amphibians | 15 | 2-3 |
| Lobe-finned fish | 19 | 2 |
| Ray-finned fish | 14 | 1-3 |
| Cartilaginous fish | 12 | 2 |

**Table S2: Number of residues, arginines and lysines in the N-terminal region of the class II SLRP member PRELP at the taxonomic class level.**

| Taxonomic class | Number of residues in the N-terminal region | Number of arginines in N-terminal region | Number of lysines in N-terminal region | Number of arginines + lysines in N-terminal region |
|---|---|---|---|---|
| Mammals | 47-52 | 4-9 | 0-4 | 7-9 |
| Birds | 39-42 | 4-5 | 1-2 | 6 |
| Reptiles | 36-40 | 3-4 | 2-3 | 6-7 |
| Amphibians | 43 | 5 | 3 | 8 |
| Lobe-finned fish | 43 | 7 | 0 | 7 |
| Ray-finned fish | 45-53 | 4-7 | 3-6 | 9-10 |
| Cartilaginous fish | 46-61 | 2-4 | 2-10 | 6-12 |

**Table S3: Number of residues and tyrosines in the N-terminal regions of the class III SLRPs mimecan, epiphycan and opticin at the taxonomic class level.**

| Taxonomic class | Number of residues in the N-terminal region | Number of tyrosines in N-terminal region |
|---|---|---|
| **Mimecan** | | |
| Mammals | 66-80 | 2-6 |
| Birds | 67-78 | 2-3 |
| Reptiles | 72-76 | 2-5 |
| Amphibians | 65-70 | 1-3 |
| Lobe-finned fish | 82 | 3 |
| Ray-finned fish | 39-71 | 1-2 |
| Cartilaginous fish | 69-77 | 2 |
| **Epiphycan** | | |
| Mammals | 93-98 | 3-5 |
| Birds | 88-92 | 3-5 |
| Reptiles | 92-93 | 3-5 |
| Amphibians | 94 | 4-5 |
| Lobe-finned fish | 101 | 5 |
| Ray-finned fish | 91-178 | 4-7 |
| Cartilaginous fish | 102 | 4-6 |
| **Opticin** | | |
| Mammals | 97-109 | 0-5 |
| Birds | 104-108 | 6 |
| Reptiles | 107-110 | 5-6 |
| Amphibians | 99 | 6 |
| Lobe-finned fish | 115 | 5 |
| Ray-finned fish | 93-105 | 4-6 |
